# Ribosomal RNA Degradation (RNA Disruption) in Tumour Cells: Mechanistic Insights and Potential Clinical Utility

**DOI:** 10.3390/cancers17172769

**Published:** 2025-08-25

**Authors:** Amadeo M. Parissenti, Sanaa Noubir, Laura B. Pritzker, Thomas Kovala, Carita Lannér, Jennifer Lemon, Tunde Onayemi, Sreepriya Pk, Gabriel Thériault, Maureen E. Trudeau, Michael M. Untch

**Affiliations:** 1 School of Natural Sciences, Laurentian University, Sudbury, ON P3E 2C6, Canada; tkovala@nosm.ca (T.K.); spk@laurentian.ca (S.P.); 2 Health Sciences North Research Institute, Sudbury, ON P3E 2H3, Canada; 3 Rna Diagnostics, Inc., Toronto, ON M4T 1L9, Canada; snoubir@rnadiagnostics.com (S.N.); lpritzker@rnadiagnostics.com (L.B.P.); jlemon@rnadiagnostics.com (J.L.); tonayemi@rnadiagnostics.com (T.O.); gtheriault@rnadiagnostics.com (G.T.); 4 Division of Medical Sciences, Northern Ontario School of Medicine, Sudbury, ON P3E 2C6, Canada; clanner@nosm.ca; 5 Odette Cancer Centre, Sunnybrook Health Sciences Centre, Toronto, ON M4N 3M5, Canada; maureen.trudeau@sunnybrook.ca; 6 Department of Obstetrics and Gynecology, Interdisciplinary Breast Cancer Center, Medical School Berlin, Helios Kliniken Berlin-Buch, 13125 Berlin, Germany; michael.untch@helios-gesundheit.de

**Keywords:** ribosomal RNA, RNA disruption, cancer, mechanisms, breast cancer, neoadjuvant chemotherapy, response biomarker, predictive biomarker

## Abstract

The ribosome in eukaryotic cells is a macromolecular complex composed of four ribonucleic acids and over 80 proteins. This organelle facilitates protein synthesis in cells, and its activity is strongly upregulated in human cancers. Immune cells, a variety of cellular stressors and numerous structurally and mechanistically distinct anti-cancer agents have been shown to induce ribosomal RNA degradation in tumour cells in vitro and in vivo—a phenomenon we termed “RNA disruption”. RNA disruption can be quantified in cultured cell lines and patient samples using the RNA disruption assay (RDA). Unlike well-known high-throughput anti-cancer drug sensitivity assays, RDA can distinguish between dying and arrested tumour cells, making it an attractive assay for anti-cancer drug discovery and development. Low tumour RNA disruption during neoadjuvant chemotherapy (as measured using RDA) is strongly associated with residual disease and reduced disease-free survival, making it a potentially valuable chemo-resistance assessment tool. High RNA disruption may also indicate chemo-responsiveness. RDA holds the prospect of being a useful tool to escalate or de-escalate neoadjuvant chemotherapy in cancer patients. Moreover, the assay’s ability to predict treatment outcomes during neoadjuvant chemotherapy may permit its use in adaptive clinical trials and in drug approval by regulatory agencies. This review provides insight into the cellular processes involved in chemotherapy-induced RNA disruption. It also describes the results of clinical studies on tumour RNA disruption in cancer patients and suggests possible approaches that could be considered for the utilization of RDAs in the clinical management of breast cancer patients undergoing current neoadjuvant chemotherapy regimens.

## Simple Summary

Ribosomes are protein-producing macromolecular complexes in mammalian cells that contain a nucleic acid called ribosomal RNA. Chemotherapy drugs and other cancer treatments can induce the degradation of ribosomal RNA in tumour cells, a phenomenon termed RNA disruption. Extensive treatment-induced RNA disruption is associated with tumour cell death and predicts both complete tumour destruction and improved disease-free survival in cancer patients. This review provides insight into the mechanisms by which various cancer treatments can induce RNA disruption. It also proposes how outcome prediction by measuring the extent of tumour RNA disruption during treatment may be used to optimize the care of patients with breast cancer by providing evidence to support treatment escalation, de-escalation, or modification decisions.

## 1. Scope of This Review

This review describes the phenomenon of RNA disruption, whereby chemotherapy agents, immune cells, and environmental stressors induce the degradation of ribosomal ribonucleic acid (rRNA) in tumour cells. RNA disruption can occur in cultured tumour cells in the laboratory (in vitro) and in the tumours of cancer patients (in vivo). Section 2 and Section 3 of this review discuss the role of the ribosome in cells and how ribosomal activity is altered during tumourigenesis, while Section 4, Section 5, Section 6, Section 7, Section 8 and Section 9 focus on the biology of RNA disruption and provide insight into possible cellular mechanisms involved in this phenomenon. Section 10 describes clinical studies that document the association between tumour RNA disruption during chemotherapy and patient outcomes, while Section 11 and Section 12 provide an overview of how on-treatment tumour RNA disruption measurements (if validated as an independent predictor of response outcome) may facilitate treatment escalation/de-escalation strategies in breast cancer and may impact adaptive clinical trials and drug approval processes. Clinicians reading this review may wish to proceed directly to Section 10.

## 2. The Ribosome: Its Composition and Roles in Mammalian Cells

The ribosome is a macromolecular complex that plays very diverse roles in cells, in particular the translation of mRNAs into proteins. Specifically, the human 80S ribosome contains over 80 different ribosomal proteins (RPs) and four ribosomal RNAs (rRNAs). It has a large 60S subunit (comprised of approximately 46 proteins and the 28S, 5.8S, and 5S rRNAs) and a small 40S subunit (comprised of approximately 33 proteins and the 18S rRNA) [1]. The catalytic activity of the ribosome is carried out by the rRNAs, while RPs residing on the surface of the ribosome stabilize its structure. Ribosomes exist as individual ribosomes in the cytoplasm of cells or as polyribosomes attached to intracellular membranes associated with the rough endoplasmic reticulum [2]. These polyribosomes form complexes with messenger RNAs (mRNAs) to translate them into proteins [3]. The ribosome serves as the site for peptide bond formation between the various amino acids that comprise a particular protein. Each three-letter codon on an mRNA pairs with the complementary anticodon of a specific tRNA. After the transcription of a particular protein-coding gene, the transcript is processed and modified, after which the resulting mRNA translocates from the nucleus to the cytoplasm [4]. The smaller subunit of the ribosome then attaches to the 5′ end of the mRNA and scans the 5′-UTR (5′ untranslated region) in the 5′→3′ direction towards the start codon (AUG) [5]. Once the 40S ribosomal RNA of the small subunit of the ribosome recognizes the start codon of the mRNA, it adds the tRNA for methionine. Subsequently, the larger 60S subunit binds to the charged 40S subunit to form the mature 80S ribosome [6]. This then initiates the translation of the transcript into a protein in the decoding centre of the ribosome [6]. The process of protein translation is regulated by a variety of regulatory factors, including eukaryotic initiation factors, elongation factors, and release factors for translation termination [7]. miRNAs and RNA-binding proteins are also involved in the process of protein translation [7,8].

## 3. Ribosomal Modifications in Cancer

In healthy cells, ribosomes ensure precise and efficient protein production according to cellular metabolic demands, which are influenced by environmental cues and nutrient availability. To maintain cellular homeostasis, ribosomal activity is tightly regulated through complex mechanisms involving signalling pathways such as mTOR (mechanistic target of rapamycin). This protein integrates nutrition availability and energy status to regulate the level of protein synthesis [9,10]. In cancer, which is characterized by uncontrolled cell proliferation, the demand for protein synthesis increases dramatically to support cell division, invasion, and metastasis. Thus, ribosome biogenesis and activity are highly increased in cancer cells [11]. This dysregulated protein synthesis often arises from genetic mutations, such as those affecting the expression or activity of tumour suppressor genes or oncogenes. For example, mutations resulting in increased *Myc* expression and/or activity during oncogenesis elevate protein synthesis rates by modulating cellular levels of specific molecules that regulate or are directly involved in protein synthesis, including RPs, translation initiation factors, RNA polymerase III, and ribosomal DNA [12]. Moreover, additional studies suggest that the hyperactive translation machinery further promotes tumourigenesis through its effects on other cellular pathways that augment cell growth, cell cycle progression, and genome instability (reviewed in [12]). Similarly, *Ras* and *Akt* mutations promote protein translation by activating the mTOR pathway (reviewed in [13]). mTOR, in turn, promotes ribosome biogenesis through its ability to regulate RNA polymerases I and III, which transcribe rRNA genes and influence rRNA processing [14,15]. *Ras* and *Akt* mutations and modifications also lead to the formation of modified ribosomes reprogrammed to preferentially translate mRNAs that code for oncogenic proteins [16,17]. Such ribosomes have been termed “onco-ribosomes”. The role of onco-ribosomes in promoting cancer stemness, immune evasion, treatment resistance, and metastasis has recently been reviewed [17].

## 4. Ribosomal RNA Fragmentation by Multiple Agents in Cell Lines

The generation of ribosomes and protein translation are among the most energy-consuming of all cellular processes, in particular since 85% of total RNA and 50% of proteins in a cell can be found in ribosomes [18]. Thus, cells adapt to the metabolic demands of stressors (such as nutrient deprivation and oxidative stress) by dramatically reducing protein translation. This involves the activation of stress-activated kinases, which block translation initiation and promote the translation of stress-responsive proteins [19]. Translational arrest can result in ribosome stalling, which can activate ribosome-associated quality control (RQC) processes that attempt to rescue and recycle stalled ribosomes [20]. Prolonged stress further results in stress granule formation and tRNA cleavage [19]. Moreover, cells adjust their numbers of ribosomes during stress through a process called ribophagy [21], in particular if the stress induces damage to RPs and mutations in ribosomal RNAs (rRNAs) [22]. Consequently, the chemotherapy treatment of tumour cells promotes rRNA degradation.

The first report of rRNA degradation mediated by chemical entities occurred more than 30 years ago by Houge et al., where the team described the ordered degradation of rRNA in a rat myeloid leukaemia cell line (IPC-81 cells) following the induction of apoptosis by cAMP analogues [23]. Treatment of IPC-81 cells with cycloheximide, actinomycin D, 7-deaza-adenosine, or calyculin A induced both rRNA degradation and apoptotic cell death [24]. In the same study, IPC-81 cells treated with cold shock, sodium azide, or hydrogen peroxide also exhibited rRNA degradation. This research group also treated NB4 human leukaemia cells with okadaic acid, primary rat thymocytes with prednisolone, and primary bovine endothelial cells with TNF/cycloheximide. All of the treatments induced apoptosis with concurrent rRNA fragmentation. The study further demonstrated that the RNA fragments obtained during treatment were derived from the 28S rRNA. It was also able to identify the specific cleavage sites using 28S-rRNA-specific probes. Taken together, these findings indicate that rRNA fragmentation occurs in multiple cell types following the induction of apoptosis mediated by various toxins [24]. Reports of apoptosis-associated rRNA fragmentation in oat cells treated with victorin and in yeast cells exposed to hydrogen peroxide, acetic acid, hyperosmotic stress, or natural aging further indicated that stressors other than toxins were able to induce rRNA fragmentation [25,26]. The fragmentation of rRNA also accompanied apoptosis in RAW 264.7 macrophages when treated with various ribotoxins [27,28]. In contrast, the induction of ribophagy in yeast by nitrogen starvation led to the bulk degradation of ribosomes, producing nucleosides, but not the ordered fragmentation of rRNAs [29]. In addition, the iron-dependent ordered fragmentation of yeast rRNA was observed following oxidative stress, which suggested a chemically induced fragmentation process independent of cell death pathways [30]. While these studies demonstrated that rRNA is often degraded into specific fragments upon exposure to toxins or environmental stressors, they did not provide quantitative data to compare the amounts of rRNA fragmentation induced by various agents. They also did not assess whether a specific level of rRNA fragmentation was necessary to be associated with cell death.

## 5. The RNA Disruption Assay (RDA) and the RNA Disruption Index (RDI)

To quantify the degree of rRNA degradation that takes place in biological samples in response to external agents, the RNA disruption assay (RDA) was developed [31]. This assay involves extracting total RNA from cells or tissues, adding a fluorescent RNA-binding dye, and resolving the various RNAs via capillary electrophoresis. This permits quantification of the various RNAs and RNA fragments in the sample and the computation of the degree of “disruption” in the normal RNA banding pattern that takes place when cells are exposed to a particular toxin or stress. The output after capillary electrophoresis is a series of RNA bands (similar to what would be seen through agarose gel electrophoresis) or a series of RNA peaks of various sizes on the RNA electropherogram. The data points defining the various peaks are then exported to calculate an RNA Disruption Index (RDI) score using a proprietary algorithm developed by RNA Diagnostics Inc. The RDI is calculated by dividing the sum of the areas of the abnormal peaks on the electropherogram by the sum of the areas of the 28S and 18S rRNAs. We have observed that on the Agilent 2100 Bioanalyzer, the abnormal peaks appear in the “inter-region” between the 28S and 18S rRNA peaks or below the 18S rRNA peak. RNA samples with high levels of intact 18S and 28S rRNAs and low levels of abnormal peaks generate low RDI values, while RNA samples with low levels of the 18S and 28S rRNAs and high levels of abnormal peaks generate high RDI values.

## 6. Chemotherapy- and Stress-Induced RNA Disruption in Tumour Cells and Its Association with Cell Death

To quantify the extent of rRNA degradation (RNA disruption) that occurs in cells and tissues in response to chemical agents, we compared the degree of RNA disruption induced by various classes of chemotherapy agents in tumour cell lines from different tissues. Narendrula et al. [32] showed that multiple structurally distinct chemotherapy agents could induce RNA disruption in a variety of breast and ovarian tumour cell lines, as shown through dose- and time-dependent increases in the RDI. Chemotherapy drugs shown to induce RNA disruption included the microtubule-binding agents paclitaxel, docetaxel, and vinblastine, the anthracyclines doxorubicin and epirubicin, the platinating agents cisplatin and carboplatin, and the topoisomerase inhibitors etoposide and irinotecan [32]. Interestingly, the degree of RNA disruption appeared to reflect cellular drug sensitivity, since docetaxel-resistant and carboplatin-resistant tumour cells did not exhibit docetaxel- or carboplatin-induced RNA disruption, respectively [32]. Moreover, drug-induced RNA disruption was associated with the cleavage of specific caspases and with caspase activity, suggesting that RNA disruption was associated with apoptotic cell death [32].

It was subsequently discovered that the cultures used in the prior study [32] had background contamination with *Mycoplasma*. We thus repeated the prior study with original stock cultures free of *Mycoplasma* (confirmed via quantitative PCR) and further assessed the breadth of RNA disruption in cell lines. Interestingly, we confirmed that a variety of chemotherapy agents were able to induce RNA disruption in tumour cells in a dose- and time-dependent manner [33]. However, the discrete fragments of rRNA observed in the prior study were not seen [33]. Rather, the RNA banding pattern in *Mycoplasma*-free cell lines resembled the banding patterns that we observed in the tumours of cancer patients undergoing neoadjuvant chemotherapy [31]. Dose- and time-dependent increases in cellular RDI values were seen in various cells, including breast epithelial cells, ovarian endometrioid cells, myeloid cells, and melanocytes [33]. However, our recent studies suggest that, in general, certain chemotherapy agents like the anthracyclines doxorubicin and epirubicin have a stronger capacity to induce RNA disruption than other chemotherapy agents, such as the platinating agents cisplatin and carboplatin or the taxanes paclitaxel and docetaxel [33]. Moreover, Northern blotting experiments with 28S-rRNA-specific DNA probes showed that the diffuse bands of rRNA fragments generated during chemotherapy-induced RNA disruption stem largely from the 28S rRNA [33]. Our studies also revealed that RNA disruption can be induced in response to specific cell stressors, including endoplasmic reticulum stress, oxidative stress, and nutrient/growth factor limitations [33]. Finally, the investigation revealed a strong relationship between the induction of RNA disruption and cell death, as measured by the loss of cell replicative capacity and the generation of cells with a subG1 DNA content [33].

We also observed, in our in vitro studies, that a variety of chemotherapy agents and cellular stressors dramatically reduced the amount of RNA isolated from cells [33]. This may be through the ability of these drugs and stressors to induce reactive oxygen species (ROS)-mediated DNA strand breaks, which result in the activation of ATM (ataxia-telangiectasia mutated) and the recruitment of NBS1 (Nijmegen Breakage Syndrome protein 1) to the nucleolus, resulting in the inhibition of ribosomal DNA transcription and ribosome biosynthesis [34]. Although a reduction in rRNA synthesis (and a consequent reduction in cellular rRNA levels) would be expected by chemotherapy agents that promote cell cycle arrest, the RNA levels in cells treated with high doses of chemotherapy agents were found to be considerably lower than untreated cells, suggesting that the reduced rRNA levels may also be due to rRNA degradation and/or cell death. The presence of rRNA fragments in the “inter-region” of the electropherogram and on Northern blots with 28S-specific DNA probes confirms that the 28S rRNA is degraded by chemotherapy agents.

Our in vitro studies have also shown a direct link between the induction of RNA disruption and cell death. However, we have also reported that little change in cellular RDI values is observed when cells are incubated with toxic agents that induce cell cycle arrest but not cell death [35]. For example, the cell-cycle-arresting agent cycloheximide was found to induce strong reductions in cell replicative capacity, colony formation, mitochondrial respiration, and cellular plasma membrane integrity at concentrations that did not induce statistically significant increases in the RDI [35]. In contrast, statistically significant increases in RDI values were consistently associated with increases in reliable biomarkers associated with cell death. These included reductions in the number of cells below pre-treatment levels and increases in the number of cells with a non-viable, subG1 DNA content [33]. Under such conditions, these cells also lost replicative capacity [33]. Table 1 summarizes in vitro studies on RNA disruption and their major findings.

## 7. The RNA Disruption Assay as a Tool for Anti-Cancer Drug Discovery

Many of the current drug sensitivity assays used to screen for prospective novel anti-cancer agents quantify the effect of the agents on specific processes associated with cell proliferation. These often serve as proxies of cell viability. For example, the MTT [3-(4,5-dimethylthiazol-2-yl)-2,5-diphenyltetrazolium bromide] or cell counting kit 8 (CCK-8) assays measure the ability of cellular dehydrogenases in living cells to reduce a tetrazolium salt to a water-soluble formazan dye. The amount of formazan produced is thus directly proportional to the number of active cells in the culture or sample [37]. Anti-cancer agents that block cell proliferation or kill cells would be expected to reduce the yield of formazan in the culture. Similarly, the clonogenic assay identifies potential anti-cancer agents based on their ability to suppress the replication of tumour cells in a semi-solid medium [38]. The latter is considered the gold standard for the detection of anti-cancer agents, due to its higher sensitivity and it being less affected by the cell plating density [39]. Nevertheless, both assays are unable to distinguish between growth-arrested (but otherwise viable) cells and non-viable/dead cells. Consequently, many of the agents identified using these assays promote transient cell cycle arrest or cell senescence, without inducing the death of tumour cells. Such surviving arrested cells may then serve as the source of drug-resistant recurrent tumours, promoting disease progression [40,41]. Consequently, compounds selected based on the results of these assays may only be cytostatic, transiently halting or slowing tumour growth, without tumour eradication. Dye exclusion assays rapidly identify agents that induce a loss of plasma membrane integrity, but such cells can remain fully viable and resume replication after drug removal [35]. Given these findings, an assay that could reliably identify agents inducing tumour cell death would potentially serve as a better tool for anti-cancer drug discovery. In 2020, Mapletoft et al. [35] compared the RDA, clonogenic, CCK-8, and Trypan blue exclusion assays for their ability to identify anti-cancer agents in human A2780 ovarian tumour cells. The study found that RNA disruption in response to chemotherapy agents occurred almost exclusively when doses were high enough such that total cell numbers decreased. An interesting additional finding was observed when A2780 cells were treated with the drug cycloheximide. Cycloheximide has been reliably used at concentrations between 0.1 mg/mL and 1 mg/mL (0.35 to 3.5 mM) to synchronize cells in the G1 phase of the cell cycle, with low effects on cell viability [42,43]. In contrast, concentrations of cycloheximide > 1.0 mM have been shown to reliably induce cell death mediated by apoptosis in mammalian cells [44,45]. The Mapletoft et al. study showed that cycloheximide was able to induce reductions in the drug sensitivity parameters of the CCK-8, clonogenic, and Trypan blue exclusion assays at concentrations used to synchronize cells without losing cell viability (<1 mM). In contrast, the RDI value of cells only increased when the dose of cycloheximide reached levels known to induce apoptosis (>1 mM) [35]. This suggests that RDA uniquely identifies dying tumour cells, but not cells undergoing cell cycle arrest. The study further found that RDA can differentiate between drug-sensitive and drug-resistant tumour cells and can identify agents capable of circumventing drug resistance. These findings provide strong evidence that RDA may be a superior drug discovery tool, specifically identifying agents that promote the death (but not arrest) of tumour cells.

## 8. Induction of Tumour RNA Disruption by Immune Cells

Beyond the ability of chemotherapy agents and cellular stressors to induce RNA disruption in vitro [32,33,35], immune cells such as natural killer (NK) cells have also been shown to induce RNA disruption in tumour cells [36]. This represents a critical mechanism in the cellular response to malignancy, whereby specialized immune cell populations actively target and destroy tumour cells. NK cells develop from bone marrow [46], common lymphoid progenitor cells [47], the liver, and the thymus [48], passing through different stages of maturation, expansion, and the acquisition of specific receptors. Similar to cytotoxic T cells, NK cells eliminate target tumour cells through two primary mechanisms: one involves the release of granules containing perforin and granzymes, while the other relies on interactions between death ligands and their corresponding death receptors [48], although both cells have distinct activation mechanisms.

NK-cell-mediated tumour RNA disruption was demonstrated in a study by Pascheto et al., in which primary NK cells were incubated with K562 chronic myeloid leukaemia cells in vitro [36]. The study showed that NK cells purified from peripheral blood mononuclear cells (PBMCs) from healthy human volunteers induced the following phenomena in K562 cells: RNA disruption (as measured based on an RDA), cytotoxicity (as measured based on the loss of plasma membrane integrity), and cell death (indicated by high levels of K562 cells with a sub-G1 DNA content) [36]. NK cells were confirmed to induce the phenomena, since PBMCs cleared of NK cells using CD56 beads did not induce RNA disruption, nor did they exhibit any cytotoxicity towards the K562 cells (Jennifer Lemon, unpublished data). Interestingly, pre-activation of the NK cells with IL-2 or pre-treatment of K562 cells with the chemotherapy drug doxorubicin significantly amplified NK cell-induced RNA disruption and cell death in K562 cells. Intriguingly, the banding patterns of RNA degradation observed in NK-cell-treated and doxorubicin-treated K562 cells were remarkably similar, suggesting that RNA disruption is a strong indicator of tumour cell death, regardless of the specific death-inducing stimulus.

One potential mechanism that may drive RNA disruption in tumour cells mediated by NK cells is the generation of ROS in tumour cells induced by granzymes, which NK cells release [49]. ROS generation can cause the oxidation of mitochondrial permeability transition pore proteins (mPTPs), leading to the release of pro-apoptotic factors and ultimately inducing tumour cell death [50]. Taken together, the study by Pascheto et al. suggests that tumour RNA disruption could serve as a valuable biomarker for assessing the efficacy of immunotherapies, including combination regimens employing both cytotoxic chemotherapy drugs and either immune cells or immunomodulatory drugs.

The ability of immune cells to induce RNA disruption has significant clinical implications. Immune-mediated RNA degradation could act synergistically with RNA-targeting chemotherapeutic agents, potentially enhancing tumour eradication. However, further investigation is required to delineate the molecular pathways underlying immune cell-induced RNA disruption and to assess its therapeutic potential across diverse tumour types. This phenomenon opens avenues for combining immunotherapy with RNA-focused strategies, potentially offering novel strategies for enhancing existing immunotherapeutic approaches.

## 9. Mechanistic Insights into Chemotherapy- and Stress-Induced RNA Disruption

The causal and contributory roles of oxidative stress and oxidative damage to cancer development and progression are well-established. ROS-induced DNA damage is known to result in mutations that drive oncogenesis through base modifications, DNA strand breaks, and DNA cross-linking [51,52]. Elevated levels of ROS increase mutations in genes involved in cell cycle regulation, apoptosis, and DNA repair, resulting in the activation of oncogenes and impaired function of tumour suppressors [52,53]. Chronic oxidative stress is also known to promote a pro-inflammatory microenvironment, which supports cancer cell proliferation, invasion, and metastasis [54,55]. Stressors can activate pathways such as NF-kB and MAPK, causing the release of inflammatory cytokines from tumour cells and the tumour microenvironment that promote tumour cell survival and metastasis [52]. The increased metabolism necessary to support the enhanced proliferation of cancer cells also results in increased ROS production, which further promotes inflammation and disease progression [56].

It is broadly accepted that most chemotherapeutic drugs induce ROS production and ROS-mediated cell injury [56,57]. Cancer cells, due to high metabolic rates and mitochondrial dysfunction, typically operate under elevated basal intracellular ROS levels, making them more vulnerable to further increases in ROS. Many chemotherapy agents leverage this vulnerability by pushing ROS levels beyond the threshold that cancer cells can manage, leading to the large-scale oxidative damage of critical cellular components, mitochondrial dysfunction, and activation of intrinsic apoptotic pathways [52,56,58]. Anthracyclines, including doxorubicin, epirubicin, and daunorubicin, generate the highest cellular ROS levels, exerting their cytotoxic effects in part by inducing extensive ROS-mediated oxidative damage to cellular organelles and molecules. If unrepairable, this results in cell death [56,57,59,60]. Platinum coordination complexes, alkylating agents, arsenic agents, and topoisomerase inhibitors also induce high levels of ROS [60,61,62], while taxanes, vinca alkaloids, antimetabolites, and nucleotide analogues also generate ROS, but at lower levels [57,60]. We have observed that several of the above chemotherapy agents induce significant ROS production (in particular, the anthracyclines) and that the ROS production is temporally correlated with the onset of RNA disruption (Samantha Ligi, unpublished observations).

The rapid production of ROS by immune cells, like the respiratory burst in neutrophils, is crucial for innate immunity functions [63]. Infections, injuries, and autoimmune disorders can trigger an exaggerated immune response, resulting in the release of large amounts of ROS. While acute oxidative stress augments the killing of invading pathogens, chronic oxidative stress can compromise the function of immune cells, including T cells, B cells, and dendritic cells. While this compromises host anti-tumour immune responses [55,64], the elevated levels of ROS production in the tumour microenvironment (combined with increased ROS production in tumour cells in response to chemotherapy agents) results in sufficient damage to promote tumour cell death.

### 9.1. ROS-Induced Damage to RPs and Mutations in rRNAs

Oxidative damage impacts the ribosome in multiple ways. This includes damaging RPs, rRNAs, and associated factors—thus impairing the ribosome’s ability to accurately assemble polypeptides [65]. Since rRNA comprises the structural and functional core of ribosomes, ROS generated in cells exposed to chemotherapy agents can significantly alter rRNA through modifications of base and sugar moieties, the generation of abasic sites, and RNA strand breaks [66]. Studies have demonstrated that both the folded rRNA structure and protein interactions do not protect rRNA from oxidative damage in the ribosome [65]. In vivo studies have indicated that rRNA in the large ribosomal subunit, including the catalytic centres, contain areas of increased susceptibility to oxidation, affecting protein synthesis, most severely under elevated oxidative stress [65,66,67].

Oxidative damage to specific RPs, such as RPS26 and RPL10, can render them non-functional. Cells mitigate this by employing dedicated chaperones—Tsr2 for RPS26 and Sqt1 for RPL10—that recognize and extract the oxidized proteins from ribosomes [68]. These chaperones facilitate the replacement of damaged proteins with newly synthesized ones, effectively repairing the ribosome [69]. This repair mechanism is crucial for maintaining ribosome numbers, particularly as ribosome assembly is downregulated during oxidative stress. Without this repair pathway, cells exhibit severely impaired growth under oxidative conditions.

Oxidative damage can lead to misfolded or dysfunctional proteins, reduced translation efficiency, and in severe cases, RP degradation via the proteasome [62,66,70]. This dysfunction may contribute to cellular stress, disrupted cellular homeostasis, and the development of diseases, including cancer [71]. Additionally, oxidative damage to ribosomes can also trigger cellular stress responses, such as the activation of autophagy and the unfolded protein response, further impacting overall cellular functions [66].

The impact of oxidative damage to ribosomes and its effect on cell functioning is still not fully understood. There likely exists a spectrum of oxidative modifications of ribosomes owing to the complexity of oxidative stress, with variations in the type and location of ROS generation, intensity and duration of oxidative stress, and the primary cellular targets [52,66]. A fascinating theory proposes that, under conditions of moderate oxidative stress, localized oxidative modifications of rRNA and RPs may facilitate adaptive processes. The end consequence is that the functionality of the total cellular ribosomal pool could separate into heterogeneous subpopulations that, under stress, function in coordination with the local cellular requirements. Considerable experimental challenges need to be overcome before this hypothesis can be tested.

Cellular redox defences counteract excess ROS under physiological conditions, minimizing damage to ribosomes and permitting normal protein translation. Evidence indicates that low-level oxidative stress drives primarily reversible modifications in rRNA and RPs [67,72]. These modifications likely facilitate cellular adaptive responses by promoting the selective translation of stress-response proteins [73]. Since translation activities in the ribosome depend on numerous precisely coordinated conformational changes and movements within the rRNA framework, oxidation of the bases that are critical for maintaining the correct rRNA structure may impair ribosome functions [66]. Highly elevated or chronic oxidative stress induces increased damage to both ribosomes and RNA, contributing to increased translational errors, stalled translation, or synthesized proteins that fail to fold correctly. Stalled transcripts on ribosomes appear to rapidly trigger the activation of both no-go decay (NGD) and RQC processes, which identify aberrant RPs [72]. The ubiquitination of RPs is the predominant mechanism by which dysfunctional RPs are targeted for degradation via the proteasome [74,75]. Consistent with this view, we have observed that a variety of RPs undergo degradation prior to or co-incident with the induction of RNA disruption mediated by chemotherapy agents and cellular stressors (Sreepriya Pk, unpublished observations).

### 9.2. ROS-Induced Activation of the Unfolded Protein Response (UPR) and RNA Disruption

We have observed that, like doxorubicin, the endoplasmic reticulum stress-inducing agents thapsigargin and tunicamycin can promote strong RNA disruption [33]. These endoplasmic reticulum stressors, as expected, also activated the unfolded protein response (UPR), as measured based on XBP1 cleavage (Sophie Branconnier, unpublished observations). Interestingly, the UPR inhibitor tauroursodeoxycholic acid (TUDCA) inhibited thapsigargin- and tunicamycin-induced XBP1 cleavage but augmented thapsigargin- and tunicamycin-induced RNA disruption. The activation of the UPR pathway is known to play a protective role in cells undergoing endoplasmic reticulum stress, but if that stress remains unresolved, UPR activation ultimately leads to cell death. TUDCA also augmented doxorubicin-induced RNA disruption (Sophie Branconnier, unpublished observations). This further supports the protective role of the UPR in inhibiting RNA disruption. While the data demonstrates that, as expected, the initial activation of the UPR pathway is a mechanism to maintain cellular homeostasis, when unresolved, it can lead to cell death and can also accelerate the rate of RNA disruption.

While the mitochondria are well established as a generator of ROS [76], the endoplasmic reticulum is estimated to be responsible for approximately 25% of ROS generated by a cell [77]. Within the endoplasmic reticulum, protein folding, disulfide bond formation and post-translational modifications occur. This process generates H_2_O_2_ as a byproduct, leading to relatively high endogenous ROS levels [78,79]. Protein folding requires both high levels of calcium to support the necessary chaperones and a highly oxidizing environment to facilitate disulfide bond formation [80,81]. Protein disulfide isomerases (PDIs) are a family of chaperones containing both oxidoreductase and isomerase activities, which play central roles in disulfide bond formation. The reoxidation of PDI is a necessary step for continued activity and involves the endoplasmic reticulum oxidoreductase 1 (ERO1), which in the process generates H_2_O_2_. A second set of enzymes also deoxidizes PDI, serving as antioxidants to reduce the ERO1-generated H_2_O_2_ to H_2_O [82,83,84]. The endoplasmic reticulum quality-assurance system ensures that improperly folded proteins are directed to the ubiquitin-proteosome system for degradation (reviewed in [85,86]). As a protective measure, UPR is initiated in cells under stress to limit overall protein expression while upregulating the expression of specific proteins that help facilitate protein folding [87,88,89]. These processes help to maintain cellular homeostasis and prevent cell death in cells under oxidative stress. However, when the endoplasmic reticulum stress is prolonged without a resolution, the misfolded proteins can act intracellularly as ligands that activate death receptor 5 (DR5), resulting in apoptotic cell death [90]. This helps to explain our observation that agents that activate the UPR (thapsigargin and tunicamycin) can promote both tumour cell RNA disruption and tumour cell death.

The UPR is a complex system regulated by three endoplasmic reticulum membrane bound receptors, inositol requiring enzyme-1 (IRE1), protein kinase R-like endoplasmic reticulum kinase (PERK), and activating transcription factor 6 (ATF6) (reviewed in [87,88,89]). Anchored in the endoplasmic reticulum membranes, these receptors are maintained in an “off” state by binding to chaperone glucose-regulated protein 78 (GRP78). GRP78 binds to unfolded proteins, competitively displacing GRP78 from the receptors, thereby activating them [91]. IRE1a activation of its RNase activity promotes the alternative splicing of X-box binding protein 1 (XBP1) mRNA, resulting in the production of the active transcription factor XBP1s. XBP1s then stimulates the transcription of specific proteins that promote protein folding and the elimination of unfolded proteins. The IRE1a RNase activity also targets RNAs within the cell for cleavage via the regulated IRE-dependent decay (RIDD) process, which degrades mRNA destined for the endoplasmic reticulum [92,93]. Initially, when pathway activation is modest, the RNA degradation is specific but becomes less so as endoplasmic reticulum stress increases and the RIDD Lacking Endomotif (RIDDLE) process is activated, producing widespread RNA degradation [94]. The potential effects of this system on rRNA degradation remain to be examined.

ROS-induced modifications of ribosomal RNAs or proteins can alter or completely inhibit ribosome function [66]. As discussed earlier, ROS also induce cell death and RNA degradation via several mechanisms. Extensive endoplasmic reticulum stress results in the generation of ROS, UPR activation, modification of ribosomal function, and eventually cell death. While the direct mechanisms connecting UPR to rRNA degradation remain to be elucidated, the pathways overlap in intriguing ways involving ROS, the UPR pathway, and RNA degradation.

### 9.3. ROS-Induced Activation of Nonfunctional RNA Decay (NRD) and Ribophagy Pathways

**Figure 1:**
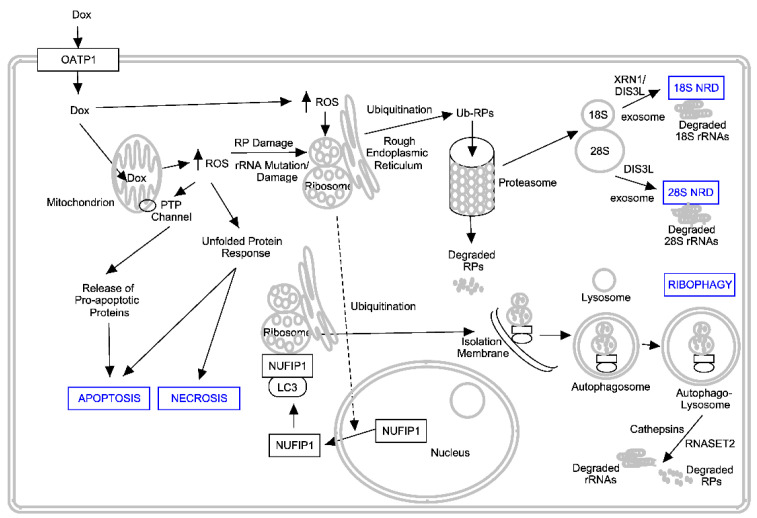
Possible mechanisms associated with the promotion of 28S/18S NRD, ribophagy, RNA disruption, and cell death mediated by the chemotherapy agent doxorubicin: doxorubicin enters the cell via diffusion and the organic anion transporter OATP1 [104]. The drug then induces high levels of reactive oxygen species (ROS) production, which results in modifications and mutations in ribosomal RNAs and damage to ribosomal proteins (RPs) [65,66]. RP damage activates the unfolded protein response (UPR) [21]. Significantly damaged RPs that cannot fold properly during UPR are subjected to degradation via the proteasome [66,70]. The ROS-induced mutations in rRNA can also promote the degradation of the 28S and 18S rRNAs through activation of the RNases (XRN1 and DIS3L) associated with the 18S and possibly 28S nonfunctional RNA decay (NRD) pathways [22]. In addition, the ROS induce the ubiquitination of RPs, a known trigger for ribosome-associated quality control (RQC), and the degradation of ribosomes mediated by ribophagy [21], possibly by activating the translocation of NUFIP1 to ribosomes and the formation of ribosome-containing autophagolysosomes. This results in both RP and rRNA hydrolysis via autophagolysosomal cathepsins and RNase T2, respectively [101]. Unresolved endoplasmic reticulum stress induced by doxorubicin’s ability to induce the production of ROS promotes the oxidation of mitochondrial permeability transition pores (mPTPs), leading to the release of pro-apoptotic factors and other proteins, ultimately inducing tumour cell death [95].

The above-described strong association of RNA disruption with tumour cell death may be due to an equally strong association between the generation of high ROS levels by chemotherapy agents in cells and the activation of a variety of pathways associated with rRNA degradation and cell death. For example, ROS can oxidize permeability transition pore (PNP) channels within mitochondria, resulting in the release of a variety of pro-apoptotic factors [50,95]. In addition, protein oxidation within the endoplasmic reticulum can induce the unfolded protein response [96], which left unchecked, activates pro-apoptotic pathways [97]. For example, as stated previously, the misfolded proteins can act intracellularly as ligands that activate death receptor 5 (DR5), resulting in apoptotic cell death [90]. High ROS levels can also promote RP degradation mediated by the proteasome [66,70] and activate autophagy [98]. If the latter is sustained, cells can die via autophagic death [99,100]. Ribophagy (the selective autophagic degradation of mature ribosomes) has also been associated with cell death induced by ROS-promoting agents [101], although most studies involved the starvation of cells as the ROS inducer and not exposure to ROS-inducing chemotherapy agents [102,103]. Interestingly, reductions in nutrients and growth factors via dilution of the cell culture medium with phosphate-buffered saline promoted strong RNA disruption in our recent studies [33]. ROS can also induce the ubiquitination of specific RPs, a known trigger for ribosome-associated quality control (RQC) and the degradation of ribosomes mediated by ribophagy [21]. The latter process may be initiated by activating the translocation of NUFIP1 to ribosomes and the formation of ribosome-containing autophagolysosomes. This results in both RP and rRNA hydrolysis via autophagolysosomal cathepsins and RNase T2, respectively [101]. ROS-induced mutations in the rRNAs have also been shown to promote degradation of the 28S and 18S rRNAs through the activation of RNases (XRN1 and DIS3L) associated with the 18S and possibly 28S nonfunctional RNA decay (NRD) pathways [22]. The transient activation of the NRD and ribophagy pathways may permit cells to survive exposure to cellular stressors and chemotherapy agents by reducing the energetically costly process of protein translation. Nevertheless, the prolonged activation of these pathways would be expected to promote cell death through organelle destruction and the activation of cell death pathways. Figure 1 depicts possible ROS-dependent mechanisms by which the chemotherapy agent doxorubicin induces both cell death and RNA disruption in tumour cells through the activation of the NRD and ribophagy pathways. It is likely that other agents and stressors known to induce RNA disruption utilize similar mechanisms.

We propose that immune cells and other chemotherapy drugs or cellular stressors also activate the above mechanisms through their common ability to induce high levels of ROS in tumour cells.

## 10. RNA Disruption as a Biomarker to Predict Outcomes from Neoadjuvant Chemotherapy

The phenomenon of RNA disruption was first discovered using total RNA preparations isolated from tumour biopsies taken from consenting patients with locally advanced breast cancer enrolled in the NCIC-CTG-MA.22 clinical trial. Patients were treated in the neoadjuvant setting with various doses of epirubicin and docetaxel at two or three weekly intervals. Ultrasound-guided core biopsies were taken from tumours prior to, during, and after treatment. Low RNA integrity in mid-treatment tumour biopsies was found to be associated with a pathologic complete response (pCR) post-treatment (one way ANOVA. *p* = 0.01) [105]. The RNA disruption assay was then developed using RNA electrophoretic data from the trial, and associations between the maximum level of tumour RNA disruption (maximum RDI value) and clinical outcome were assessed. High mid-treatment tumour RNA disruption (RDI > 35) correlated with pCR at surgery; seven out of eight patients with a pCR at surgery had RDI values > 35, whereas none of the patients with RDI values < 10 experienced a pCR [106]. Subsequent Kaplan–Meier analyses revealed that patients with high RNA disruption (RDI > 50) had significantly higher disease-free survival (DFS) durations than patients with tumour RDI values < 10 (hazard ratio 2.0; Mantel Cox test *p* = 0.05) [31]. Increased RNA disruption in response to chemotherapy was observed in all breast cancer subtypes. Interestingly, DFS durations were equivalent between patients with high RNA disruption (n = 38) and patients that achieved a pCR at surgery (n = 8) (mean DFS durations of 56.9 ± 5.6 months versus 59.4 ± 12.8 months). The higher number of patients identified as chemotherapy responders using RNA disruption assessments suggests that RDA may be a better predictor of survival than pCR. Moreover, the data suggests that high RNA disruption may predict improved survival independently of pCR.

No correlation with pCR or DFS was found in RNA isolated from pre-therapy or post-therapy tumour biopsies. Since tumour RNA disruption occurs in response to therapy, the use of pre-therapy biopsies for outcome prediction based on RDAs would be counter-intuitive. RDA tests on post-therapy tumour biopsies showed little prediction value, likely because the effect of treatment on tumour cell RNA may have waned as the tumour bed is repopulated with viable stromal and immune cells. The strong association of RNA disruption with tumour cell death in vitro (Section 6) may help explain the utility of RDA in predicting treatment outcomes in cancer patients, provided that the treating agent induces tumour cell death in vivo.

Understanding if RNA disruption occurs as a treatment response in diseases outside of breast cancer was explored in a prospective clinical trial of canine lymphoma at the Ontario Veterinary College [107]. Dogs (n = 41) treated with CHOP chemotherapy (cyclophosphamide, doxorubicin, vincristine, prednisone) had fine-needle aspirate samples taken before treatment and at 3, 6, and 11 weeks post-treatment. RNA was isolated, and RNA disruption was assessed. High RNA disruption was found to correlate with improved progression-free survival (PFS); the median PFS for dogs with an average tumour RDI > 0.6 was 9.1 months compared with 4.9 months for dogs with average tumour RDI values ≤ 0.6 (log-rank test *p* = 0.006) [107]. This study suggests that on-treatment tumour RNA disruption measurements may be useful in predicting outcomes from neoadjuvant chemotherapy in patients with a variety of cancers [107].

Further examination into the variety of drug combinations that can lead to RNA disruption was performed using retrospective data taken from an Ireland Clinical Oncology Research Group TCHL study (NCTO1485926) and the NeoAva clinical trial (NCT00773695). The TCHL study was a phase II neoadjuvant trial assessing pCR rates in patients receiving docetaxel, carboplatin, and trastuzumab ± lapatinib where core biopsies were taken 20 days after one cycle of therapy. RNA disruption was assessable in tumour biopsies taken at this earlier timepoint and with these drug combinations. RNA disruption correlated with response to therapy; RDI scores were increased in patients who had a pCR at surgery (n = 7) compared with patients who had a partial or no response (n = 10) (10.2 ± 5.1 versus 5.4 ± 2.2; *p* = 0.025) [108].

RNA disruption caused by the angiogenesis inhibitor bevacizumab was explored using retrospective data from the NeoAva clinical trial, a neoadjuvant study of 132 eligible patients with early primary HER2- breast cancer. Patients received epirubicin, 5-fluorouracil, and cyclophosphamide (FEC) for 12 weeks followed by taxane therapy (paclitaxel or docetaxel) for 12 weeks. Half of the patients (66) also received concurrent bevacizumab for 24 weeks. The co-administration of bevacizumab increased the pCR rate [defined as residual cancer burden (RCB) class 0] from 5 to 20% in patients with ER+ tumours [109]. However, the increased pCR rate was only associated with an improved DFS in strong responders to treatment (patients have a RCB class of 0 or 1) [109]. RNA disruption was assessable in 98 of the 12 week samples; the median tumour RDI value for responders to treatment (patients in RCB classes 0 or 1) was significantly greater than that of non-responding patients in RCB class 3 (median 2.6, n = 23 versus median 1.3, n = 17, respectively; *p* = 0.006, Mann–Whitney test). Patients with tumour RDI values > 1.1 had significantly greater DFS than patients with tumour RDI values ≤ 1.1. Treatment with bevacizumab resulted in a significantly higher median tumour RDI values in the 12-week samples (*p* = 0.003, Mann–Whitney test) [109]. These two studies support our in vitro findings that treatment with a wide variety of chemotherapy agents can result in RNA disruption in tumour cells.

Differing biopsy collection and storage methods and varying RNA isolation methodologies were used in the above-described retrospective studies, resulting in a wide range of tumour RDI values among the studies. The BREVITY (**B**reast Cancer **R**esponse **Ev**aluation for **I**ndividualized **T**herap**y**) clinical trial (NCT03524430) was designed to validate the utility of on-treatment tumour RNA disruption measurements to predict outcomes from neoadjuvant chemotherapy in patients with breast cancer using standardized methodologies for tumour sample collection and storage, the homogenization of samples, and RNA isolation. BREVITY was designed to acquire two separate datasets. The training dataset from 80 patients in the trial was used to establish the range of tumour RDI values to be expected in patients with breast cancer. In addition, pCR outcome data from the training dataset allowed us to set specific RDI cut-points to define specific zones of chemotherapy responses in the patients [strong responders (zone 3), moderate responders (zone 2), and non-responders to treatment (zone 1)]. As reported by Cazzaniga et al. [110], in the BREVITY training set, maximum RDI values for pCR responders were significantly higher than in patients without a pCR post-treatment (11.3 ± 1.6 vs. 6.8 ± 0.6; *p* = 0.008). Maximum tumour RDI values ≤ 3.7 predicted a lack of pCR with a negative predictive value (NPV) of 93.3% [110]. All assay parameters (including RDI cut points) have now been fixed in the second phase of the trial for hopeful validation in a set of 454 patients. The accrual of patients for the validation set is approximately 90% complete. A summary of translational research studies assessing the utility of RDA to predict clinical responses and outcomes from neoadjuvant chemotherapy in human and canine cancer patients can be found in Table 2.

## 11. Potential Impact of the RNA Disruption Assay on Patient Care

Drug approvals in oncology are usually based on evidence from randomized clinical trials (RCTs) with large cohorts of cancer patients. RCTs aim to demonstrate a statistically significant improvement in outcome in the group of patients treated with the investigative drug compared to the best available standard of care in the control group. A statistically significant improvement in outcome does not imply that all patients in the group treated with the new drug will have a benefit. A subgroup of these patients may endure toxicities without deriving an advantage in terms of DFS or overall survival. An important challenge is to find ways to optimize each patient’s treatment with a focus on both enhanced survival and reduced drug adverse effects and toxicities. It is critical to give patients all treatments that may enhance their survival, i.e., treatment escalation if potentially beneficial. By the same principle that seeks the best interests of the patient, a treatment that causes side effects without a potential benefit should be avoided i.e., treatment de-escalation if de-escalation does not sacrifice the potential for benefit. Treatment de-escalation may reduce both cumulative drug toxicities and financial burdens for patients, e.g., high costs of drugs and long treatment durations that may be multiple, sequential, and interdependent in administration. One example of a treatment de-escalation was the NSABP B-51 trial that showed that omitting adjuvant regional nodal irradiation in patients who had positive ipsilateral axillary nodes before neoadjuvant chemotherapy and became lymph-node-negative after neoadjuvant chemotherapy did not increase the risk of disease recurrence or death from breast cancer [111]. This de-escalation approach has shown to be safe and is now in the guidelines for the above-mentioned patient category [112].

The neoadjuvant setting may allow several de-escalation and escalation strategies because the tumour response can be assessed during drug administration as per the principle of response-guided therapy. Gepar Trio [113] was the first trial to evaluate response-guided neoadjuvant therapy with a trial design that used palpation and sonography to assess patients’ tumour response after two cycles of docetaxel, doxorubicin, and cyclophosphamide (TAC regimen). The trial randomly assigned early responders to four or six additional TAC cycles and early non-responders to four cycles of TAC or vinorelbine and capecitabine (NX regimen). While the Gepar Trio, as designed, did not result in an improvement in patient survival (for the patient group that underwent treatment adaptation)—to the extent that would support a change in treatment guidelines, the trial produced valuable insights for the future of response-guided therapy. Both adaptation strategies, i.e., extending the treatment of early responders with the same but prolonged chemotherapy and switching early non-responders to non–cross-resistant chemotherapy, improved DFS. Unplanned post hoc combined analysis showed superior survival for both response-guided treatment groups versus both conventional treatment groups. Further exploratory DFS and overall survival analyses by cancer subtype revealed that this specific strategy of the Gepar Trio trial, as designed, works particularly well in patients with HR+ tumours.

To be impactful, response-guided therapy requires (a) the reliable prediction of response with a high-performance diagnostic tool and (b) the choice of available drugs to consider as neoadjuvant treatment substitutes to increase the chances of a response and improved survival. Furthermore, the Gepar Trio trial showed that high Ki67 measured in the invasive residual tumour at surgery predicted poor prognosis [114]. The lack of further treatment possibilities to offer post-surgery to the patient group with poor prognosis emphasized the need for innovative drugs in post-neoadjuvant-treatment escalation. The experience with Ki67 has shown that while quantitative biological markers can be reliable, their standardization is often challenging, which hampers their validation as predictive or prognostic assays.

A discussion of the potential utility of RDA as a candidate predictive biomarker that may support response-guided therapy considering today’s availability of alternative drug treatments and strategies follows.

### 11.1. RDA’s Ability to Predict Tumour Response to Treatment in the Neoadjuvant Setting

Differentiating non-responders to therapy from responders with a well-validated standardized test is key to the success of response-guided therapy. The BREVITY study [115] is seeking to validate the potential use of RDA (veridapt DX™) as a predictive test of treatment efficacy. In BREVITY, RDA requires a first tumour core biopsy taken as early as 31–39 days after the initiation of a treatment A and then a second biopsy taken at 2–3 weeks after a switch to a second treatment B, to assess the overall effect of the two treatments and to generate a tumour RDI value or score. This score can be valuable in decision-making because it provides a prediction of pathological and survival outcomes before the end of treatment and surgery. Action may be taken to de-escalate or escalate treatment well before surgery (for example, three cycles before the end of a standard-of-care neoadjuvant therapy regimen). The tumour biopsy procedure required for RDA testing is performed with technologies existing in hospitals and community clinical centres. In these clinical centres, breast tumour biopsies are routinely performed for cancer diagnostic purposes. In the BREVITY trial, all logistical processes—from tumour biopsy collection to storage and shipment of biopsy material—are tightly controlled. Subsequently, biopsy homogenization, RNA isolation, and capillary electrophoresis of the RNA samples are conducted using standardized procedures in a CLIA-certified laboratory. The controlled processes established in the BREVITY trial have allowed for the standardization of RDA testing. In these standardized processes, RDA’s response zone cut-offs have been established in phase 1 of BREVITY, using a training set of 80 fully evaluable patients. As stated previously, tumour RDI values in the non-response zone (RDI ≤ 3.7) were associated with the absence of a pCR (NPV of 93.3%) [110]. If RDA’s performance in the training set is validated in an independent cohort of 452 fully evaluable patients (with an NPV > 90%), RDA may become a valuable standardized guidance test for drug escalation/de-escalation strategies. Identifying non-responding patients with >90% NPV in a timely manner during neoadjuvant treatment with a standardized molecular test would provide valuable guidance with high reliability. Furthermore, RDA testing can be performed and communicated to the treating physician within 2–3 days from the reception of the tumour biopsies. This rapid turnaround allows RDA to be actionable and integrable with current practices in the clinical management of cancer patients.

### 11.2. Today’s Availability of Alternative Drug Treatments and Strategies

The last decade has seen the discovery of several new classes of drugs that have helped to modernize approaches to breast cancer treatment and lead to changes in international treatment guidelines. Some innovative drugs (antibody drug conjugates, CDK4/6 inhibitors, PARP inhibitors, selective estrogen degraders, and immune checkpoint inhibitors) have allowed for considerable improvements in outcomes for patients with breast cancer of all subtypes. Furthermore, there are ongoing trials that are now testing new drug candidates and drug treatment combinations for further potential benefits. Most of these trials aim to find ways to optimize treatment through de-escalation and escalation strategies.

#### 11.2.1. In High-Risk Luminal B Early Breast Cancer

The standard of care for these patients includes up to eight cycles of combined taxanes and anthracyclines in the neoadjuvant setting, followed by surgery and loco-regional irradiation. Adjuvant drug treatment for these patients includes CDK4/6 inhibitors in combination with hormonal therapy. Two CDK4/6 inhibitors (abemaciclib [116] and ribociclib [117]) have been approved by the FDA and EMA in March 2023 and September 2024, respectively, following positive results from the MonarchE and NATALEE trials. These two large randomized controlled trials were mostly escalation trials because CDK4/6 inhibitors were given after neoadjuvant or adjuvant chemotherapy in the treatment of the majority of patients. However, a small percentage of patients received CDK4/6 inhibitors without previous chemotherapy. Prior to these trials, standard of care treatment was six to eight cycles of taxanes and anthracyclines, followed by 5–10 years of hormonal therapy (aromatase inhibitors or tamoxifen, with the addition of ovarian suppression in premenopausal patients). Upon their approval in high-risk cancer, CDK4/6 inhibitors may be added to hormonal therapy in the adjuvant setting following 5–6 months of treatment with chemotherapy, surgery, and irradiation. The combination of CDK4/6 inhibitors with hormonal therapy in the adjuvant setting is currently prescribed for 2–3 years, offering a DFS benefit but also contributing to cumulative toxicities. This development has led to a new paradigm question—whether chemotherapy treatment may be reduced to decrease both the high cumulative toxicity and the delay in surgery and adjuvant therapy. In high-risk luminal B breast cancer, an RDA performed during neoadjuvant chemotherapy may help predict treatment outcome. Where marginal to no benefit of additional cycles of chemotherapy is predicted, moving to surgery and adjuvant treatment with hormonal therapy and CDK4/6 inhibitors could be beneficial. The ADAPT trial led by the West German Study Group has recently finished patient accrual and will show whether the de-escalation of chemotherapy can be performed by giving endocrine therapy plus CDK4/6 inhibitors [118]. The ongoing multicentric international trial NoLEEta led by French Breast Cancer Intergroup UNICANCER has the same purpose by aiming to compare two patient study arms, one treated with chemotherapy followed by CDK4/6 inhibition and endocrine therapy and another treated with CDK4/6 inhibitors and endocrine therapy but without chemotherapy [119].

#### 11.2.2. In HER2+ Early Breast Cancer

Long-term medical follow-ups of patients treated with anthracycline-based regimens have shown serious cardiotoxicities [120] across cancer subtypes. However, despite the availability of alternative drugs that target HER2 (trastuzumab and pertuzumab), anthracyclines continue to be prescribed for patients with HER2+ breast cancer. Omitting treatment with anthracyclines may reduce survival benefit in patients whose tumours may respond to these drugs. Undertreatment may cause instances of earlier deaths. Alternative therapies that are anthracycline-free [for example, six cycles of taxotere, carboplatin, herceptin, and pertuzumab (TCHP)] may be considered, based on evidence from the TRAIN-2 trial [121,122]. An alternate drug treatment de-escalation strategy is being investigated by researchers at Shantou University, who are comparing the safety and efficacy of two combination treatments [TCHP and THP (without carboplatin)] [123]. Published findings from this study support the safety of this de-escalation approach and the non-inferiority of the THP regimen without carboplatin. The findings of the neoCARHP clinical trial recently presented at the ASCO 2025 annual meeting demonstrated identical pCR rates in the two treatment groups (non-inferiority of THP), indicating that the omission of carboplatin could further reduce toxicities without sacrificing outcome (pending long-term survival data in the specific patient population participating in the trial) [123,124]. An unmet need that will persist despite these positive results is to identify, during neoadjuvant therapy, which patients would need treatment with platinum compounds and which patients may be safely spared from their toxicities, since carboplatin is still widely used in the treatment of patients with HER2 overexpressing tumours. Options of treatment de-escalations may be adopted to further reduce risk of undertreatment when supported by response-prediction tests. If validated in the BREVITY clinical trial, RDA’s ability to identify patients who are highly unlikely to obtain a pCR and to predict enhanced DFS (a secondary endpoint of BREVITY) may provide critical decision support for this group of patients.

The KATHERINE study is an escalation trial that demonstrated a survival benefit of trastuzumab emtansine (TDM-1) given after surgery in HER2+ patients with no-pCR after neoadjuvant chemotherapy combined with anti-HER2 targeted therapy. DESTINY Breast-05 (a joint study by the GBG, AGO-B, NSABP, and SOLTI research groups) examines the efficacy of trastuzumab deruxtecan (T-DXd) compared with T-DM1 in high-risk patients with residual invasive breast cancer following neoadjuvant therapy [125]. Another escalation strategy may employ Neratinib (an irreversible pan-HER tyrosine kinase inhibitor) for patients who do not achieve a pCR after neoadjuvant therapy. The ExTeNET trial has shown a significant survival benefit of Neratinib in patients with HR+/HER2+ tumours who had residual disease after neoadjuvant therapy [126,127,128]. Non-PCR (high risk) HER2+ patients—treated with Neratinib less than 1 year from their last dose of trastuzumab and fully completing Neratinib treatment—showed 5-year invasive DFS and 8-year overall survival benefits of 11.9% and 13.2%, respectively [126,127].

In the forefront for treatment de-escalation, several trials are currently exploring the use of dual HER2 blockade (trastuzumab and pertuzumab) to identify and adapt treatment based on an early response to therapy, ultimately aiming to reduce or eliminate chemotherapy. The trials DECRESCENDO, DESTINY-Breast11, and PHERGAIN-2 are currently underway to test the potential benefit of the de-escalation of anthracyclines or omitting chemotherapy entirely and treating patients instead with HER2-targeted drugs (trastuzumab/pertuzumab) and/or the antibody drug conjugates TDM-1 and T-DXd [129,130,131]. The PHERGAIN 1 trial showed that, while ^18^fluorine-fluorodeoxyglucose positron emission tomography (FDG-PET) alone was ineffective at independently identifying responders to neoadjuvant trastuzumab/pertuzumab (HP) treatment in patients with early HER2+ breast cancer, about a third of responders to HP therapy (identified by FDG-PET) who also achieved a pCR post-treatment were able to forgo adjuvant TCHP chemotherapy. pCR was, by far, the superior indicator of a response to HP treatment compared to FDG-PET in this trial. Nevertheless, the combined strategy did identify patients that could forgo adjuvant chemotherapy with carboplatin and docetaxel with an excellent invasive DFS at 3 years and fewer treatment-related adverse events [132]. PHERGAIN-2 is using imaging methods to assess tumour response in the context of these de-escalation decisions [133]. As an alternative or in addition to an assessment with imaging methods, laboratory standardized RDA testing could provide a drug-response prediction that relies on biological analyses of the tumour (rather than solely on imaging, which performs poorly as a response predictor). The combined response-assessment approach may allow the greater reliability of prediction of non-pCR in these critical de-escalation decisions.

#### 11.2.3. In Triple Negative Early Breast Cancer (TNBC)

Currently, the standard of care in the neoadjuvant setting for patients with triple negative early breast cancer is neoadjuvant chemotherapy plus the immune checkpoint inhibitor drug pembrolizumab (a drug that can make tumours more susceptible to destruction by the immune system). The KEYNOTE-522 trial [134,135,136] supported the escalation of treatment, as it showed that the addition of pembrolizumab to neoadjuvant chemotherapy significantly increased the percentage of patients with TNBC who achieved a pCR. Patients who received neoadjuvant pembrolizumab plus chemotherapy, followed by adjuvant pembrolizumab after surgery, had a significantly longer event-free survival and overall survival than patients who received neoadjuvant chemotherapy alone [134,135,136]. Ongoing clinical trials are evaluating strategies of further escalation to improve outcomes for patients with TNBC that fail to achieve a pCR following neoadjuvant chemotherapy plus pembrolizumab. Several new drug candidates and drug treatment combinations are being tested in the SASCIA, ASCENT-05, MK2870-012, and Tropion Breast 04 trials [137,138,139]. These trials aim to determine whether escalation with novel antibody drug conjugates (sacituzumab govitecan, sacituzumab tirumotecan, and datopotamab deruxtecan) and employing innovative treatment combinations with chemotherapy, targeted therapy, and immune checkpoint inhibitors will provide incremental benefits. In the context of these treatment-escalation approaches, an RDA may predict non-pCR during standard of care treatment with chemotherapy plus pembrolizumab and allow an earlier addition or switch to an alternative drug. Patients with non-responding tumours may also be considered for enrolment in ongoing clinical trials and a priority access to investigative drugs. As such, RDA, as a reliable predictor of treatment outcome/benefit (provided the RDA is validated in phase 2 of BREVITY), may help accelerate investigations into new drugs and drug approvals. Tumour response prediction during neoadjuvant therapy guided by an RDA may allow drug-escalation strategies before surgery is performed and pathology results are made available.

In the NeoSTAR trial [140], de-escalation has been attempted employing response-guided therapy in the neoadjuvant setting. Patients with TNBC received 12 weeks of neoadjuvant treatment with pembrolizumab and sacituzumab govitecan, and tumour response to these drugs was evaluated by imaging to detect the presence or absence of residual disease. As presented at the June 2025 ASCO meeting, in 24 patients with no residual disease suspected based on imaging, pathology results at surgery showed residual vital cancer cells in 8 patients (1/3 of the patients). This means that neoadjuvant treatment in these patients would have been stopped, based on the assumption that they had a complete response. In contrast, 9 out of 26 patients that had suspected residual disease (~1/3) were found to have no residual vital cancer cells at surgery after additional neoadjuvant chemotherapy per investigator choice. This means that additional neoadjuvant chemotherapy with added toxicity would have occurred in two thirds of the patients, with a questionable added benefit in terms of the pathological outcome. These findings from the NeoSTAR trial illustrate the importance of selecting tumour-response-assessment methods with greater performance and reliability than palpation, ultrasound, mammography, and even MRI or PET-CT. If RDA is validated in phase 2 of the BREVITY trial for prediction of non-pCR with an NPV > 90% and for prediction of enhanced DFS in patients with high RDA scores, RDA may provide valuable tumour response guidance that could help to mitigate the risks of treatment escalation or de-escalation.

## 12. Future Perspectives, Including Use of RDA in Adaptive Clinical Trials and Drug Approval Processes

**Figure 2:**
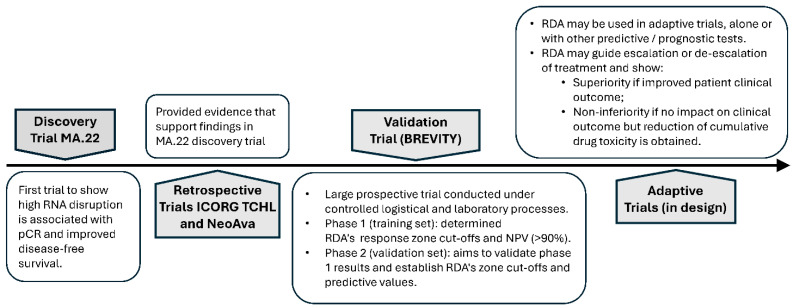
Development and evolution of RDA in clinical studies in early breast cancer. The MA.22 (discovery trial) showed that high tumour RNA disruption during treatment was associated with a high pCR rate and improved DFS [31]. The patient population (N = 85) in the MA.22 trial included all breast tumour subtypes. Further retrospective studies, ICORG TCHL (N = 17) and NeoAva (N = 98), supported the MA.22 trial findings in two breast cancer patient subgroups: HER2+ [108] and HER-, respectively [109]. BREVITY is a large prospective trial conducted in two phases to validate the prediction utility of RDA in tightly controlled logistical processes from tumour biopsy collection to biopsy storage, shipment, and transport. In the BREVITY clinical trial, an RDA is performed using standardized procedures in a CLIA-certified laboratory. Phase 1 of BREVITY (training set of 80 patients with early breast cancer of all tumour subtypes) has met its primary objective to determine RDA’s response zone cut-offs and NPV in terms of the pCR. Tumour RDI values ≤ 3.7 predicted a lack of pCR with an NPV of 93.3% [110]. The larger cohort of patients (n = 452) in the validation phase of the BREVITY clinical trial seeks to confirm established RDA performance characteristics, i.e., NPV and PPV in terms of the prediction of pCR and/or disease-free survival. Patient enrolment for the validation phase of BREVITY is currently 90% complete. Subsequently, studies will investigate RDA’s clinical utility in each breast cancer subtype. In the future, the RDA may be incorporated into adaptive clinical trial designs, alone or in combination with other drug-response-assessment methods, to compare the effectiveness of response-guided treatment with adaptation to that of a specific standard of care without adaptation. Adaptive trials may evaluate several response-guided treatment approaches and test their effectiveness in terms of (i) superiority if an improved patient clinical outcome in terms of pCR and/or DFS is obtained or (ii) the ability to reduce cumulative drug toxicities while demonstrating non-inferiority in terms of clinical outcome.

As described in Section 10 and illustrated in Figure 2, chemotherapy-dependent rRNA degradation (RNA disruption) and its quantification was first described in a retrospective study associated with the NCIC-CTG MA.22 clinical trial [31]. While the trial provided compelling evidence that high RNA disruption mid-treatment was associated with an increased pCR rate and improved DFS after neoadjuvant chemotherapy, the number of patients assessed was small with no pre-defined assessment parameters. Nevertheless, two additional retrospective studies supported these original findings, including studies associated with the ICORG TCHL clinical trial [108] and the NeoAva clinical trial [109]. To provide level 1 evidence to support RDA’s utility to predict treatment outcome, a prospective study (BREVITY) involving a much larger patient cohort (n > 450) with pre-defined endpoints was initiated. The BREVITY training set of patients used to set and fix the assay parameters for subsequent validation supported the prior findings by showing significantly higher on-treatment tumour RNA disruption in pCR responders than patients with residual disease [110]. Moreover, low tumour RNA disruption during treatment was found to be associated with residual disease post-treatment with an NPV of 93.3% [110]. In the BREVITY validation set of patients, it is hoped that level 1 evidence can be provided to support RDA’s utility as a tool to predict treatment outcomes early or during neoadjuvant chemotherapy, regardless of the drug regimen employed, including those involving immunomodulatory drugs [115]. Should the predictive utility of the RDA be validated in the large BREVITY cohort, subsequent analyses will examine the predictive clinical utility of the RDA across tumour subtypes. RDAs could also be subsequently used as a treatment-outcome-prediction tool in adaptive clinical trials. This would permit adjusting patient accrual in the various arms of a clinical trial, based on real-time assessments of drug responses using the RDA. The assay could also be used to support superiority or non-inferiority claims for certain treatment protocols. As suggested in Section 11, RDA, if validated, could be used alone or in combination with other predictive biomarkers to guide treatment escalation or de-escalation strategies. This could include an assessment of changes in circulating tumour DNA (ctDNA) levels in response to neoadjuvant chemotherapy, although recent studies supporting utility have involved small patient cohorts with specific tumour subytpes, particularly TNBC [141]. Finally, should mid-treatment RNA disruption assessments prove superior to post-treatment pCR assessments in predicting improved DFS, RDA (alone or in combination with other predictive biomarkers) could be used as a reliable surrogate of treatment outcomes for drug approval by governmental regulatory agencies.

## 13. Conclusions

A variety of mechanistically distinct chemotherapy agents, immune cells, and environmental stressors induce RNA disruption in tumour cells. The ability of these treatments to induce RNA disruption may be related to their common ability to induce the production of reactive oxygen species, which induce damage and/or mutations in DNA, rRNAs, proteins, and RPs in tumour cells. Unresolved stress and damage could then activate specific pathways associated with cell death and rRNA degradation in tumour cells, including ribophagy, non-functional RNA decay, and apoptosis. Clinically, high mid-treatment tumour RNA disruption was found to be associated with a higher pCR rate and/or improved DFS after neoadjuvant chemotherapy in both human breast cancer patients and canine lymphoma patients. The ability of a quantitative standardized RNA disruption assay to predict pCR and improved DFS in patients with breast cancer is being rigorously assessed in an ongoing multinational clinical trial (BREVITY). Should the extent of tumour RNA disruption be validated as an independent predictor of treatment outcome in this trial, the RNA disruption assay may help oncologists optimize the treatment of breast cancer by providing valuable response data to facilitate treatment escalation, de-escalation, or modification.

## Figures and Tables

**Table 1:** Summary of recent in vitro studies on RNA disruption in tumour cells.

Study	Cell Lines	Agents Inducing rRNA Degradation/ RNA Disruption	Study Findings	Reference
Houge et al. (1993)	Rat Myeloid Leukaemia (IPC-81)	cAMP analogues	Apoptosis induction by cAMP analogues induces cleavage of 28S rRNA at the V3 and V13 variable regions	[23]
Houge et al. (1995)	Rat Myeloid Leukaemia (IPC-81)	cycloheximide actinomycin D 7-deasa-adenosine calyculin A sodium azide hydrogen peroxide	Fine mapping of 28S rRNA cleavage sites using a variety of apoptosis-inducing agents	[24]
Houge et al. (1995)	Human Leukaemia (NB4); Primary rat thymocytes; Primary bovine endothelial cells	okadaic acid prednisolone tumour necrosis factor alpha cycloheximide	A wide variety of apoptosis-inducing cytotoxic agents induce 28S rRNA cleavage in a variety of cell lines	[24]
Hoat et al. (2006)	Oat plant	Victorin	Specific cleavages of rRNA and mRNA occur during victorin-induced apoptosis in oat cells	[25]
Mroczek and Kufel (2008)	Yeast cells	hydrogen peroxide acetic acid hyperosmotic agents	Stressors other than toxins can also induce rRNA fragmentation	[26]
He et al. (2012)	RAW 264.7 murine macrophages	deoxynivalenol	Mycotoxin deoxynivalenol-induce apoptosis is accompanied by specific cleavages of the 28S rRNA through activation of specific signal transduction pathways	[27]
He et al. (2012)	RAW 264.7 murine macrophages	ribotoxins	Four ribotoxins induce p53-dependent rRNA cleavage via activation of cathepsin L and caspase-3	[28]
Huang et al. (2015)	Yeast cells	Nitrogen starvation	Nitrogen starvation induces autophagy and bulk RNA degradation in yeast	[29]
Narendrula et al. (2016)	Human A2780 ovarian tumour cells; Human CaOV3 ovarian tumour cells Human MDA-MB-231 breast tumour cells	docetaxel paclitaxel carboplatin cisplatin doxorubicin epirubicin etoposide vinblastine irinotecan	Multiple chemotherapy agents induce rRNA degradation (RNA disruption) in human tumour cell lines	[32]
Zinskie et al. (2018)	Yeast cells	iron oxidative stress	Iron-dependent cleavage of rRNA during oxidative stress independent of cell death pathways	[30]
Pascheto et al. (2020)	K562 chronic myeloid leukaemia cells	NK cells	NK cells induce RNA disruption and cell death in myeloid leukaemia cells	[36]
Mapletoft et al. (2022)	Human A2780 ovarian tumour cells	cycloheximide doxorubicin	The RNA disruption assay (RDA) is superior to various drug sensitivity assays in detecting cytotoxic drugs	[35]
Butler et al. (2023)	Human A2780 ovarian tumour cells; Human MDA-MB-231 breast tumour cells; Human K562 chronic myeloid leukemic cells; Human A375 melanoma cells; Normal human vascular endothelial cells (HUVECs); Normal murine NiH 3T3 fibroblast cells; Normal human MCF-10A breast epithelial cells	doxorubicin epirubicin etoposide cisplatin carboplatin paclitaxel docetaxel vinblastine irinotecan palbociclib thapsigargin tunicamycin cycloheximide nutrient limitation hydrogen peroxide	A wide variety of structurally and mechanistically distinct chemotherapy agents and several cellular stressors induce RNA disruption in multiple tumour cell lines—yielding similar RNA-degradation patterns. High RNA disruption is associated with cell death	[33]

**Table 2:** Summary of recent clinical studies on tumour RNA disruption in cancer patients.

Study	Type	Number of Patients	Disease	Findings	Reference
MA.22	Retrospective	85	Breast Cancer—all subtypes	High RNA disruption associated with pCR and improved disease-free survival	[31]
ICORG TCHL	Retrospective	17	HER2+ Breast Cancer	High RNA disruption associated with pCR	[108]
NeoAva	Retrospective	98	HER2- Breast Cancer	High RNA disruption associated with disease-free survival	[109]
OVC Canine Lymphoma	Prospective	41	Canine Lymphoma	High RNA disruption associated with progression-free survival	[107]
BREVITY Training Set	Prospective	80	Breast Cancer—all subtypes	Low RNA disruption associated with pCR absence	[110]

## Abbreviations

The following abbreviations are used in this manuscript:

ANOVA	analysis of variance
ATM	ataxia-telangiectasia mutated
CCK-8	cell counting kit 8
CDK	cyclin-dependent kinase
DDR	DNA damage repair
DFS	disease-free survival
EMT	epithelial mesenchymal transition
ER	estrogen receptor
FDG-PET	18fluorine-fluorodeoxyglucose positron emission tomography
FEC	5-fluoro uracil, epirubicin, and cyclophosphamide
GRP78	glucose regulated protein 78
HER2	human epidermal growth factor receptor 2
Ki67	cellular proliferation marker
miRNA	micro ribonucleic acid
MRI	magnetic resonance imaging
mRNA	messenger RNA
mTOR	molecular target of rapamycin
MTT	3-(4,5-dimethylthiazol-2-yl)-2,5-diphenyltetrazolium bromide
NGD	no go decay
NK	natural killer
NPV	negative predictive value
NRD	non-functional RNA decay
NX	vinorelbine and capecitabine
PARP	poly ADP-ribose polymerase
pCR	pathologic complete response
PCR	polymerase chain reaction
PET-CT	positron emission tomography–computed tomography
PFS	progression-free survival
PR	progesterone receptor
RCB	residual cancer burden
RCT	randomized clinical trial
RDA	RNA disruption assay
RDI	RNA disruption index
ROS	reactive oxygen species
RNA	ribonucleic acid
RP	ribosomal protein
RQC	ribosome quality control
rRNA	ribosomal ribonucleic acid
T	Taxane
TAC	taxotere, adriamycin, and cyclophosphamide
TCHP	taxotere, carboplatin, herceptin, and pertuzumab
TDM1	trastuzumab emtansine
T-DXd	trastuzumab deruxtecan
THP	taxotere, herceptin and pertuzumab
TNBC	triple negative breast cancer
TNF	tumour necrosis factor
tRNA	transfer ribonucleic acid
TUDCA	tauroursodeoxycholic acid
UPR	unfolded protein response
XBP1	X-box binding protein 1

## References

[1] Wilson D.N., Doudna Cate J.H. (2012). The structure and function of the eukaryotic ribosome. Cold Spring Harb. Perspect. Biol..

[2] Thomson E., Ferreira-Cerca S., Hurt E. (2013). Eukaryotic ribosome biogenesis at a glance. J. Cell Sci..

[3] Afonina Z.A., Myasnikov A.G., Shirokov V.A., Klaholz B.P., Spirin A.S. (2015). Conformation transitions of eukaryotic polyribosomes during multi-round translation. Nucleic Acids Res..

[4] Paci G., Caria J., Lemke E.A. (2021). Cargo transport through the nuclear pore complex at a glance. J. Cell Sci..

[5] Sharifulin D., Khairulina Y., Ivanov A., Meschaninova M., Ven’Yaminova A., Graifer D., Karpova G. (2012). A central fragment of ribosomal protein S26 containing the eukaryote-specific motif YxxPKxYxK is a key component of the ribosomal binding site of mRNA region 5′ of the E site codon. Nucleic Acids Res..

[6] Andreev D.E., O’Connor P.B., Loughran G., Dmitriev S.E., Baranov P.V., Shatsky I.N. (2017). Insights into the mechanisms of eukaryotic translation gained with ribosome profiling. Nucleic Acids Res..

[7] Jia X., He X., Huang C., Li J., Dong Z., Liu K. (2024). Protein translation: Biological processes and therapeutic strategies for human diseases. Signal Transduct. Target. Ther..

[8] Bastide A., David A. (2018). Interaction of rRNA with mRNA and tRNA in Translating Mammalian Ribosome: Functional Implications in Health and Disease. Biomolecules.

[9] Kim S.G., Buel G.R., Blenis J. (2013). Nutrient regulation of the mTOR complex 1 signaling pathway. Mol. Cells.

[10] Rosario F.J., Powell T.L., Gupta M.B., Cox L., Jansson T. (2020). mTORC1 Transcriptional Regulation of Ribosome Subunits, Protein Synthesis, and Molecular Transport in Primary Human Trophoblast Cells. Front. Cell Dev. Biol..

[11] Elhamamsy A.R., Metge B.J., Alsheikh H.A., Shevde L.A., Samant R.S. (2022). Ribosome Biogenesis: A Central Player in Cancer Metastasis and Therapeutic Resistance. Cancer Res..

[12] Ruggero D. (2009). The role of Myc-induced protein synthesis in cancer. Cancer Res..

[13] Grzmil M., Hemmings B.A. (2012). Translation regulation as a therapeutic target in cancer. Cancer Res..

[14] Mayer C., Grummt I. (2006). Ribosome biogenesis and cell growth: mTOR coordinates transcription by all three classes of nuclear RNA polymerases. Oncogene.

[15] Michels A.A. (2011). MAF1: A new target of mTORC1. Biochem. Soc. Trans..

[16] Bastide A., David A. (2018). The ribosome, (slow) beating heart of cancer (stem) cell. Oncogenesis.

[17] Ramalho S., Dopler A., Faller W.J. (2024). Ribosome specialization in cancer: A spotlight on ribosomal proteins. NAR Cancer.

[18] Scott M., Gunderson C.W., Mateescu E.M., Zhang Z., Hwa T. (2010). Interdependence of cell growth and gene expression: Origins and consequences. Science.

[19] Advani V.M., Ivanov P. (2019). Translational Control under Stress: Reshaping the Translatome. Bioessays.

[20] Vind A.C., Genzor A.V., Bekker-Jensen S. (2020). Ribosomal stress-surveillance: Three pathways is a magic number. Nucleic Acids Res..

[21] Sun M., Zhang X., Tan B., Zhang Q., Zhao X., Dong D. (2024). Potential role of endoplasmic reticulum stress in doxorubicin-induced cardiotoxicity-an update. Front. Pharmacol..

[22] Coria A.R., Shah A., Shafieinouri M., Taylor S.J., Guiblet W., Miller J.T., Mani Sharma I., Wu C.C. (2025). The integrated stress response regulates 18S nonfunctional rRNA decay in mammals. Mol. Cell.

[23] Houge G., Doskeland S.O., Boe R., Lanotte M. (1993). Selective cleavage of 28S rRNA variable regions V3 and V13 in myeloid leukemia cell apoptosis. FEBS Lett..

[24] Houge G., Robaye B., Eikhom T.S., Golstein J., Mellgren G., Gjertsen B.T., Lanotte M., Doskeland S.O. (1995). Fine mapping of 28S rRNA sites specifically cleaved in cells undergoing apoptosis. Mol. Cell. Biol..

[25] Hoat T.X., Nakayashiki H., Tosa Y., Mayama S. (2006). Specific cleavage of ribosomal RNA and mRNA during victorin-induced apoptotic cell death in oat. Plant J..

[26] Mroczek S., Kufel J. (2008). Apoptotic signals induce specific degradation of ribosomal RNA in yeast. Nucleic Acids Res..

[27] He K., Zhou H.R., Pestka J.J. (2012). Targets and intracellular signaling mechanisms for deoxynivalenol-induced ribosomal RNA cleavage. Toxicol. Sci..

[28] He K., Zhou H.R., Pestka J.J. (2012). Mechanisms for ribotoxin-induced ribosomal RNA cleavage. Toxicol. Appl. Pharmacol..

[29] Huang H., Kawamata T., Horie T., Tsugawa H., Nakayama Y., Ohsumi Y., Fukusaki E. (2015). Bulk RNA degradation by nitrogen starvation-induced autophagy in yeast. EMBO J..

[30] Zinskie J.A., Ghosh A., Trainor B.M., Shedlovskiy D., Pestov D.G., Shcherbik N. (2018). Iron-dependent cleavage of ribosomal RNA during oxidative stress in the yeast Saccharomyces cerevisiae. J. Biol. Chem..

[31] Parissenti A.M., Guo B., Pritzker L.B., Pritzker K.P., Wang X., Zhu M., Shepherd L.E., Trudeau M.E. (2015). Tumor RNA disruption predicts survival benefit from breast cancer chemotherapy. Breast Cancer Res. Treat..

[32] Narendrula R., Mispel-Beyer K., Guo B., Parissenti A.M., Pritzker L.B., Pritzker K., Masilamani T., Wang X., Lannér C. (2016). RNA disruption is associated with response to multiple classes of chemotherapy drugs in tumor cell lines. BMC Cancer.

[33] Butler P., Pascheto I., Lizzi M., St-Onge R., Lanner C., Guo B., Masilamani T., Pritzker L.B., Kovala A.T., Parissenti A.M. (2023). RNA disruption is a widespread phenomenon associated with stress-induced cell death in tumour cells. Sci. Rep..

[34] Larsen D.H., Hari F., Clapperton J.A., Gwerder M., Gutsche K., Altmeyer M., Jungmichel S., Toledo L.I., Fink D., Rask M.B. (2014). The NBS1-Treacle complex controls ribosomal RNA transcription in response to DNA damage. Nat. Cell Biol..

[35] Mapletoft J.P.J., St-Onge R.J., Guo B., Butler P., Masilamani T.J., D’Costa L., Pritzker L.B., Parissenti A.M. (2020). The RNA disruption assay is superior to conventional drug sensitivity assays in detecting cytotoxic drugs. Sci. Rep..

[36] Pascheto I., Pritzker L.B., Kumar A., Parissenti A.M. (2020). Quantification of immune cell-mediated destruction of tumor cells in vitro using the RNA disruption assay. J. Clin. Oncol..

[37] Mosmann T. (1983). Rapid colorimetric assay for cellular growth and survival: Application to proliferation and cytotoxicity assays. J. Immunol. Methods.

[38] Puck T.T., Marcus P.I., Cieciura S.J. (1956). Clonal growth of mammalian cells in vitro; growth characteristics of colonies from single HeLa cells with and without a feeder layer. J. Exp. Med..

[39] Citrin D.E. (2016). Short-Term Screening Assays for the Identification of Therapeutics for Cancer. Cancer Res..

[40] Milanovic M., Fan D.N.Y., Belenki D., Dabritz J.H.M., Zhao Z., Yu Y., Dorr J.R., Dimitrova L., Lenze D., Monteiro Barbosa I.A. (2018). Senescence-associated reprogramming promotes cancer stemness. Nature.

[41] Reimann M., Lee S., Schmitt C.A. (2024). Cellular senescence: Neither irreversible nor reversible. J. Exp. Med..

[42] Liu X., Yang J.M., Zhang S.S., Liu X.Y., Liu D.X. (2010). Induction of cell cycle arrest at G1 and S phases and cAMP-dependent differentiation in C6 glioma by low concentration of cycloheximide. BMC Cancer.

[43] Macur K., Grzenkowicz-Wydra J., Konieczna L., Bigda J., Temporini C., Tengattini S., Baczek T. (2018). A Proteomic-Based Approach to Study the Mechanism of Cytotoxicity Induced by Interleukin-1alpha and Cycloheximide. Chromatographia.

[44] Gong J., Li X., Darzynkiewicz Z. (1993). Different patterns of apoptosis of HL-60 cells induced by cycloheximide and camptothecin. J. Cell Physiol..

[45] Blom W.M., de Bont H.J., Meijerman I., Mulder G.J., Nagelkerke J.F. (1999). Prevention of cycloheximide-induced apoptosis in hepatocytes by adenosine and by caspase inhibitors. Biochem. Pharmacol..

[46] Rosmaraki E.E., Douagi I., Roth C., Colucci F., Cumano A., Di Santo J.P. (2001). Identification of committed NK cell progenitors in adult murine bone marrow. Eur. J. Immunol..

[47] Kondo M., Weissman I.L., Akashi K. (1997). Identification of clonogenic common lymphoid progenitors in mouse bone marrow. Cell.

[48] Sojka D.K., Tian Z., Yokoyama W.M. (2014). Tissue-resident natural killer cells and their potential diversity. Semin. Immunol..

[49] Prager I., Watzl C. (2019). Mechanisms of natural killer cell-mediated cellular cytotoxicity. J. Leukoc. Biol..

[50] England K., Cotter T.G. (2005). Direct oxidative modifications of signalling proteins in mammalian cells and their effects on apoptosis. Redox Rep..

[51] Hayes J.D., Dinkova-Kostova A.T., Tew K.D. (2020). Oxidative Stress in Cancer. Cancer Cell.

[52] Jelic M.D., Mandic A.D., Maricic S.M., Srdjenovic B.U. (2021). Oxidative stress and its role in cancer. J. Cancer Res. Ther..

[53] Aggarwal V., Tuli H.S., Varol A., Thakral F., Yerer M.B., Sak K., Varol M., Jain A., Khan M.A., Sethi G. (2019). Role of Reactive Oxygen Species in Cancer Progression: Molecular Mechanisms and Recent Advancements. Biomolecules.

[54] Mantovani A., Allavena P., Sica A., Balkwill F. (2008). Cancer-related inflammation. Nature.

[55] Lin Y., Jiang M., Chen W., Zhao T., Wei Y. (2019). Cancer and ER stress: Mutual crosstalk between autophagy, oxidative stress and inflammatory response. Biomed. Pharmacother..

[56] Yang H., Villani R.M., Wang H., Simpson M.J., Roberts M.S., Tang M., Liang X. (2018). The role of cellular reactive oxygen species in cancer chemotherapy. J. Exp. Clin. Cancer Res..

[57] Jiang H., Zuo J., Li B., Chen R., Luo K., Xiang X., Lu S., Huang C., Liu L., Tang J. (2023). Drug-induced oxidative stress in cancer treatments: Angel or devil?. Redox Biol..

[58] Ju S., Singh M.K., Han S., Ranbhise J., Ha J., Choe W., Yoon K.S., Yeo S.G., Kim S.S., Kang I. (2024). Oxidative Stress and Cancer Therapy: Controlling Cancer Cells Using Reactive Oxygen Species. Int. J. Mol. Sci..

[59] Doroshow J.H. (2019). Mechanisms of Anthracycline-Enhanced Reactive Oxygen Metabolism in Tumor Cells. Oxidative Med. Cell Longev..

[60] Zhang Y., Ding C., Zhu W., Li X., Chen T., Liu Q., Zhou S., Zhang T.C., Ma W. (2022). Chemotherapeutic drugs induce oxidative stress associated with DNA repair and metabolism modulation. Life Sci..

[61] Sun Y., Wang C., Wang L., Dai Z., Yang K. (2018). Arsenic trioxide induces apoptosis and the formation of reactive oxygen species in rat glioma cells. Cell. Mol. Biol. Lett..

[62] Liu J., Qu L., Meng L., Shou C. (2019). Topoisomerase inhibitors promote cancer cell motility via ROS-mediated activation of JAK2-STAT1-CXCL1 pathway. J. Exp. Clin. Cancer Res..

[63] Banerjee S., Ghosh S., Mandal A., Ghosh N., Sil P.C. (2020). ROS-associated immune response and metabolism: A mechanistic approach with implication of various diseases. Arch. Toxicol..

[64] Shah R., Ibis B., Kashyap M., Boussiotis V.A. (2024). The role of ROS in tumor infiltrating immune cells and cancer immunotherapy. Metabolism.

[65] Willi J., Kupfer P., Evequoz D., Fernandez G., Katz A., Leumann C., Polacek N. (2018). Oxidative stress damages rRNA inside the ribosome and differentially affects the catalytic center. Nucleic Acids Res..

[66] Shcherbik N., Pestov D.G. (2019). The impact of oxidative stress on ribosomes: From injury to regulation. Cells.

[67] Yan L.L., Zaher H.S. (2019). How do cells cope with RNA damage and its consequences?. J. Biol. Chem..

[68] Yang Y.M., Karbstein K. (2024). Ribosome Assembly and Repair. Annu. Rev. Cell Dev. Biol..

[69] Spiri S., Brar G.A. (2023). Fix it, don’t trash it: Ribosome maintenance by chaperone-mediated repair of damaged subunits. Mol. Cell.

[70] Muhar M.F., Farnung J., Cernakova M., Hofmann R., Henneberg L.T., Pfleiderer M.M., Denoth-Lippuner A., Kalcic F., Nievergelt A.S., Peters Al-Bayati M. (2025). C-terminal amides mark proteins for degradation via SCF-FBXO31. Nature.

[71] Simms C.L., Hudson B.H., Mosior J.W., Rangwala A.S., Zaher H.S. (2014). An active role for the ribosome in determining the fate of oxidized mRNA. Cell Rep..

[72] Yan L.L., Simms C.L., McLoughlin F., Vierstra R.D., Zaher H.S. (2019). Oxidation and alkylation stresses activate ribosome-quality control. Nat. Commun..

[73] Gerashchenko M.V., Lobanov A.V., Gladyshev V.N. (2012). Genome-wide ribosome profiling reveals complex translational regulation in response to oxidative stress. Proc. Natl. Acad. Sci. USA.

[74] Manohar S., Jacob S., Wang J., Wiechecki K.A., Koh H.W.L., Simoes V., Choi H., Vogel C., Silva G.M. (2019). Polyubiquitin Chains Linked by Lysine Residue 48 (K48) Selectively Target Oxidized Proteins In Vivo. Antioxid. Redox Signal..

[75] Dougherty S.E., Barros G.C., Foster M.W., Teo G., Choi H., Silva G.M. (2025). Context specific ubiquitin modification of ribosomes regulates translation under oxidative stress. bioRxiv.

[76] Zhao R.Z., Jiang S., Zhang L., Yu Z.B. (2019). Mitochondrial electron transport chain, ROS generation and uncoupling (Review). Int. J. Mol. Med..

[77] Tu B.P., Weissman J.S. (2004). Oxidative protein folding in eukaryotes: Mechanisms and consequences. J. Cell Biol..

[78] Araki K., Iemura S., Kamiya Y., Ron D., Kato K., Natsume T., Nagata K. (2013). Ero1-alpha and PDIs constitute a hierarchical electron transfer network of endoplasmic reticulum oxidoreductases. J. Cell Biol..

[79] Enyedi B., Várnai P., Geiszt M. (2010). Redox state of the endoplasmic reticulum is controlled by Ero1L-alpha and intraluminal calcium. Antioxid. Redox Signal..

[80] Michalak M., Robert Parker J.M., Opas M. (2002). Ca^2+^ signaling and calcium binding chaperones of the endoplasmic reticulum. Cell Calcium.

[81] Hwang C., Sinskey A.J., Lodish H.F. (1992). Oxidized Redox State of Glutathione in the Endoplasmic Reticulum. Science.

[82] Wang L., Zhang L., Niu Y., Sitia R., Wang C.C. (2014). Glutathione peroxidase 7 utilizes hydrogen peroxide generated by Ero1alpha to promote oxidative protein folding. Antioxid. Redox Signal..

[83] Ramming T., Hansen H.G., Nagata K., Ellgaard L., Appenzeller-Herzog C. (2014). GPx8 peroxidase prevents leakage of H_2_O_2_ from the endoplasmic reticulum. Free Radic. Biol. Med..

[84] Konno T., Pinho Melo E., Lopes C., Mehmeti I., Lenzen S., Ron D., Avezov E. (2015). ERO1-independent production of H_2_O_2_ within the endoplasmic reticulum fuels Prdx4-mediated oxidative protein folding. J. Cell Biol..

[85] Krshnan L., Siu W.S., Van de Weijer M., Hayward D., Guerrero E.N., Gruneberg U., Carvalho P. (2022). Regulated degradation of the inner nuclear membrane protein SUN2 maintains nuclear envelope architecture and function. elife.

[86] Christianson J.C., Carvalho P. (2022). Order through destruction: How ER-associated protein degradation contributes to organelle homeostasis. EMBO J..

[87] Klein A.M., de Queiroz R.M., Venkatesh D., Prives C. (2021). The roles and regulation of MDM2 and MDMX: It is not just about p53. Genes Dev..

[88] Read A., Schröder M. (2021). The Unfolded Protein Response: An Overview. Biology.

[89] Hetz C., Zhang K., Kaufman R.J. (2020). Mechanisms, regulation and functions of the unfolded protein response. Nat. Rev. Mol. Cell. Biol..

[90] Lam M., Marsters S.A., Ashkenazi A., Walter P. (2020). Misfolded proteins bind and activate death receptor 5 to trigger apoptosis during unresolved endoplasmic reticulum stress. elife.

[91] Bertolotti A., Zhang Y., Hendershot L.M., Harding H.P., Ron D. (2000). Dynamic interaction of BiP and ER stress transducers in the unfolded-protein response. Nat. Cell Biol..

[92] Hollien J., Weissman J.S. (2006). Decay of endoplasmic reticulum-localized mRNAs during the unfolded protein response. Science.

[93] Hollien J., Lin J.H., Li H., Stevens N., Walter P., Weissman J.S. (2009). Regulated Ire1-dependent decay of messenger RNAs in mammalian cells. J. Cell Biol..

[94] Le Thomas A., Ferri E., Marsters S., Harnoss J.M., Lawrence D.A., Zuazo-Gaztelu I., Modrusan Z., Chan S., Solon M., Chalouni C. (2021). Decoding non-canonical mRNA decay by the endoplasmic-reticulum stress sensor IRE1α. Nat. Commun..

[95] Zhou Y., Jing S., Liu S., Shen X., Cai L., Zhu C., Zhao Y., Pang M. (2022). Double-activation of mitochondrial permeability transition pore opening via calcium overload and reactive oxygen species for cancer therapy. J. Nanobiotechnol..

[96] Zhang Z., Zhang L., Zhou L., Lei Y., Zhang Y., Huang C. (2019). Redox signaling and unfolded protein response coordinate cell fate decisions under ER stress. Redox Biol..

[97] Fribley A., Zhang K., Kaufman R.J. (2009). Regulation of apoptosis by the unfolded protein response. Methods Mol. Biol..

[98] Yun H.R., Jo Y.H., Kim J., Shin Y., Kim S.S., Choi T.G. (2020). Roles of Autophagy in Oxidative Stress. Int. J. Mol. Sci..

[99] Chen Y., McMillan-Ward E., Kong J., Israels S.J., Gibson S.B. (2008). Oxidative stress induces autophagic cell death independent of apoptosis in transformed and cancer cells. Cell Death Differ..

[100] Yuan G.J., Deng J.J., Cao D.D., Shi L., Chen X., Lei J.J., Xu X.M. (2017). Autophagic cell death induced by reactive oxygen species is involved in hyperthermic sensitization to ionizing radiation in human hepatocellular carcinoma cells. World J. Gastroenterol..

[101] Kazibwe Z., Liu A.Y., MacIntosh G.C., Bassham D.C. (2019). The Ins and Outs of Autophagic Ribosome Turnover. Cells.

[102] Kraft C., Deplazes A., Sohrmann M., Peter M. (2008). Mature ribosomes are selectively degraded upon starvation by an autophagy pathway requiring the Ubp3p/Bre5p ubiquitin protease. Nat. Cell Biol..

[103] Wyant G.A., Abu-Remaileh M., Frenkel E.M., Laqtom N.N., Dharamdasani V., Lewis C.A., Chan S.H., Heinze I., Ori A., Sabatini D.M. (2018). NUFIP1 is a ribosome receptor for starvation-induced ribophagy. Science.

[104] Durmus S., Naik J., Buil L., Wagenaar E., van Tellingen O., Schinkel A.H. (2014). In vivo disposition of doxorubicin is affected by mouse Oatp1a/1b and human OATP1A/1B transporters. Int. J. Cancer.

[105] Parissenti A.M., Chapman J.A., Kahn H.J., Guo B., Han L., O’Brien P., Clemons M.P., Jong R., Dent R., Fitzgerald B. (2010). Association of low tumor RNA integrity with response to chemotherapy in breast cancer patients. Breast Cancer Res. Treat..

[106] Pritzker K., Pritzker L., Generali D., Bottini A., Cappelletti M.R., Guo B., Parissenti A., Trudeau M. (2015). RNA Disruption and Drug Response in Breast Cancer Primary Systemic Therapy. J. Natl. Cancer Inst. Monogr..

[107] Parissenti A.M., Pritzker L.B., Guo B., Narendrula R., Wang S.X., Lin L.L., Pei J., Skowronski K., Bienzle D., Woods J.P. (2019). RNA disruption indicates CHOP therapy efficacy in canine lymphoma. BMC Vet. Res..

[108] Toomey S., Eustace A.J., Pritzker L.B., Pritzker K.P., Fay J., O’Grady A., Cummins R., Grogan L., Kennedy J., O’Connor D. (2016). RE: RNA disruption assay as a biomarker of pathological complete response in neoadjuvant trastuzumab-treated human epidermal growth factor receptor 2-positive breast cancer. J. Natl. Cancer Inst..

[109] Parissenti A.M., Pritzker L.B., Dahle M.A., Gythfeldt H.V.L., Masilamani T., Theriault G., St-Onge R., D’Costa L., Lingjaerde O.C., Haugen M.H. (2025). High mid-treatment tumour RNA disruption in patients with HER2-negative breast cancer is associated with improved disease-free survival after neoadjuvant chemotherapy. Breast Cancer Res..

[110] Cazzaniga M.E., Ademuyiwa F., Petit T., Tio J., Generali D., Ciruelos E.M., Califaretti N., Poirier B., Ardizzoia A., Hoenig A. (2024). Low RNA disruption during neoadjuvant chemotherapy predicts pathologic complete response absence in patients with breast cancer. JNCI Cancer Spectr..

[111] Mamounas E.P., Bandos H., White J.R., Julian T.B., Khan A.J., Shaitelman S.F., Torres M.A., Vicini F.A., Ganz P.A., McCloskey S.A. (2025). Omitting Regional Nodal Irradiation after Response to Neoadjuvant Chemotherapy. N. Engl. J. Med..

[112] Goodman A. Some Patients with Breast Cancer May Safely Avoid Locoregional Irradiation After Neoadjuvant Chemotherapy. ASCO Post. https://ascopost.com/issues/february-25-2024/some-patients-with-breast-cancer-may-safely-avoid-locoregional-irradiation-after-neoadjuvant-chemotherapy/.

[113] von Minckwitz G., Blohmer J.U., Costa S.D., Denkert C., Eidtmann H., Eiermann W., Gerber B., Hanusch C., Hilfrich J., Huober J. (2013). Response-guided neoadjuvant chemotherapy for breast cancer. J. Clin. Oncol..

[114] Denkert C., Loibl S., Muller B.M., Eidtmann H., Schmitt W.D., Eiermann W., Gerber B., Tesch H., Hilfrich J., Huober J. (2013). Ki67 levels as predictive and prognostic parameters in pretherapeutic breast cancer core biopsies: A translational investigation in the neoadjuvant GeparTrio trial. Ann. Oncol..

[115] Trudeau M.G.D., Ademuyiwa F., Petit T., Tio J., Ciruelos E., Jankowski T. RNA Disruption Assay (RDA)-Breast Cancer Response Evaluation for Individualized Therapy (BREVITY). https://clinicaltrials.gov/study/NCT03524430.

[116] Rastogi P., O’Shaughnessy J., Martin M., Boyle F., Cortes J., Rugo H.S., Goetz M.P., Hamilton E.P., Huang C.S., Senkus E. (2024). Adjuvant Abemaciclib Plus Endocrine Therapy for Hormone Receptor-Positive, Human Epidermal Growth Factor Receptor 2-Negative, High-Risk Early Breast Cancer: Results from a Preplanned monarchE Overall Survival Interim Analysis, Including 5-Year Efficacy Outcomes. J. Clin. Oncol..

[117] Slamon D., Lipatov O., Nowecki Z., McAndrew N., Kukielka-Budny B., Stroyakovskiy D., Yardley D.A., Huang C.S., Fasching P.A., Crown J. (2024). Ribociclib plus Endocrine Therapy in Early Breast Cancer. N. Engl. J. Med..

[118] Hofmann D., Nitz U., Gluz O., Kates R.E., Schinkoethe T., Staib P., Harbeck N. (2013). WSG ADAPT-adjuvant dynamic marker-adjusted personalized therapy trial optimizing risk assessment and therapy response prediction in early breast cancer: Study protocol for a prospective, multi-center, controlled, non-blinded, randomized, investigator initiated phase II/III trial. Trials.

[119] Bidard F.C. Absence de Chimiothérapie Adjuvante Dans le Cancer du Sein Précoce HR+ HER2- à Risque Intermédiaire Traité par Ribociclib (LEE-011), un Essai de Phase III de Non-Infériorité. https://www.unicancer.fr/wp-content/uploads/2024/09/2706_ucbg_newsletter-n19.pdf.

[120] Volkova M., Russell R. (2011). Anthracycline cardiotoxicity: Prevalence, pathogenesis and treatment. Curr. Cardiol. Rev..

[121] van Ramshorst M.S., van der Voort A., van Werkhoven E.D., Mandjes I.A., Kemper I., Dezentje V.O., Oving I.M., Honkoop A.H., Tick L.W., van de Wouw A.J. (2018). Neoadjuvant chemotherapy with or without anthracyclines in the presence of dual HER2 blockade for HER2-positive breast cancer (TRAIN-2): A multicentre, open-label, randomised, phase 3 trial. Lancet Oncol..

[122] van der Voort A., van Ramshorst M.S., van Werkhoven E.D., Mandjes I.A., Kemper I., Vulink A.J., Oving I.M., Honkoop A.H., Tick L.W., van de Wouw A.J. (2021). Three-Year Follow-up of Neoadjuvant Chemotherapy with or Without Anthracyclines in the Presence of Dual ERBB2 Blockade in Patients With ERBB2-Positive Breast Cancer: A Secondary Analysis of the TRAIN-2 Randomized, Phase 3 Trial. JAMA Oncol..

[123] Wu S., Bian L., Wang H., Zhang S., Wang T., Yu Z., Li J., Li F., Wang K., Jiang Z. (2024). De-escalation of neoadjuvant taxane and carboplatin therapy in HER2-positive breast cancer with dual HER2 blockade: A multicenter real-world experience in China. World J. Surg. Oncol..

[124] Gao H.-F., Li W., Wu Z., Dong J., Cao Y., Zhao Y., Chen Q.-J., Ma S., Ouyang J., Ye J.-H. (2025). De-escalated neoadjuvant taxane plus trastuzumab and pertuzumab with or without carboplatin in HER2-positive early breast cancer (neoCARHP): A multicentre, open-label, randomised, phase 3 trial. J. Clin. Oncol..

[125] Sankyo D. A Study of Trastuzumab Deruxtecan (T-DXd) Versus Trastuzumab Emtansine (T-DM1) in High-Risk HER2-Positive Participants with Residual Invasive Breast Cancer Following Neoadjuvant Therapy (DESTINY-Breast05). https://clinicaltrials.gov/study/NCT04622319.

[126] Holmes F.A., Moy B., Delaloge S., Chia S.K.L., Ejlertsen B., Mansi J., Iwata H., Gnant M., Buyse M., Barrios C.H. (2023). Overall survival with neratinib after trastuzumab-based adjuvant therapy in HER2-positive breast cancer (ExteNET): A randomised, double-blind, placebo-controlled, phase 3 trial. Eur. J. Cancer.

[127] Martin M., Holmes F.A., Ejlertsen B., Delaloge S., Moy B., Iwata H., von Minckwitz G., Chia S.K.L., Mansi J., Barrios C.H. (2017). Neratinib after trastuzumab-based adjuvant therapy in HER2-positive breast cancer (ExteNET): 5-year analysis of a randomised, double-blind, placebo-controlled, phase 3 trial. Lancet Oncol..

[128] Chan A., Delaloge S., Holmes F.A., Moy B., Iwata H., Harvey V.J., Robert N.J., Silovski T., Gokmen E., von Minckwitz G. (2016). Neratinib after trastuzumab-based adjuvant therapy in patients with HER2-positive breast cancer (ExteNET): A multicentre, randomised, double-blind, placebo-controlled, phase 3 trial. Lancet Oncol..

[129] Zeneca A. Trastuzumab Deruxtecan (T-DXd) Alone or in Sequence with THP, Versus Standard Treatment (ddAC-THP), in HER2-Positive Early Breast Cancer. https://clinicaltrials.gov/study/NCT05113251.

[130] Piccart M. De-Escalation Adjuvant Chemo in HER2+/ER-/Node-Neg Early BC Patients Who Achieved PCR After Neoadjuvant Chemo & Dual HER2 Blockade (Decrescendo). https://clinicaltrials.gov/study/NCT04675827.

[131] Llombart A. Chemotherapy-Free Trastuzumab and Pertuzumab in HER2-Positive Breast Cancer: FDG-PET Response-Adapted Strategy. (PHERGain). https://clinicaltrials.gov/study/NCT03161353.

[132] Perez-Garcia J.M., Cortes J., Ruiz-Borrego M., Colleoni M., Stradella A., Bermejo B., Dalenc F., Escriva-de-Romani S., Calvo Martinez L., Ribelles N. (2024). 3-year invasive disease-free survival with chemotherapy de-escalation using an (18)F-FDG-PET-based, pathological complete response-adapted strategy in HER2-positive early breast cancer (PHERGain): A randomised, open-label, phase 2 trial. Lancet.

[133] Llombart-Cussac A. Chemotherapy-Free pCR-Guided Strategy with Trastuzumab-Pertuzumab and T-DM1 in HER2-Positive Early Breast Cancer (PHERGAIN-2). https://www.clinicaltrials.gov/study/NCT04733118.

[134] Schmid P., Cortes J., Pusztai L., McArthur H., Kummel S., Bergh J., Denkert C., Park Y.H., Hui R., Harbeck N. (2020). Pembrolizumab for Early Triple-Negative Breast Cancer. N. Engl. J. Med..

[135] Schmid P., Cortes J., Dent R., Pusztai L., McArthur H., Kummel S., Bergh J., Denkert C., Park Y.H., Hui R. (2022). Event-free Survival with Pembrolizumab in Early Triple-Negative Breast Cancer. N. Engl. J. Med..

[136] Schmid P., Cortes J., Dent R., McArthur H., Pusztai L., Kummel S., Denkert C., Park Y.H., Hui R., Harbeck N. (2024). Overall Survival with Pembrolizumab in Early-Stage Triple-Negative Breast Cancer. N. Engl. J. Med..

[137] Sciences G. Study of Sacituzumab Govitecan-Hziy and Pembrolizumab Versus Treatment of Physician’s Choice in Patients with Triple Negative Breast Cancer Who Have Residual Invasive Disease After Surgery and Neoadjuvant Therapy (ASCENT-05/AFT-65 OptimICE-RD/GBG 119/NSABP B-63). https://clinicaltrials.gov/study/NCT05633654.

[138] Dohme M.S. Sacituzumab Tirumotecan (MK-2870) Plus Pembrolizumab Versus TPC in TNBC Who Did Not Achieve pCR (MK-2870-012). https://clinicaltrials.gov/study/NCT06393374.

[139] McArthur H.L., Tolaney S.M., Dent R., Schmid P., Asselah J., Liu Q., Meisel J.L., Niikura N., Park Y.H., Werutsky G. (2025). TROPION-Breast04: A randomized phase III study of neoadjuvant datopotamab deruxtecan (Dato-DXd) plus durvalumab followed by adjuvant durvalumab versus standard of care in patients with treatment-naive early-stage triple negative or HR-low/HER2- breast cancer. Ther. Adv. Med. Oncol..

[140] Spring L.M. Sacituzumab Govitecan in TNBC (NeoSTAR). https://www.clinicaltrials.gov/study/NCT04230109.

[141] Chen J.H., Addanki S., Roy D., Bassett R., Kalashnikova E., Spickard E., Kuerer H.M., Meas S., Sarli V.N., Korkut A. (2024). Monitoring response to neoadjuvant chemotherapy in triple negative breast cancer using circulating tumor DNA. BMC Cancer.

[142] Kutmon M., van Iersel M.P., Bohler A., Kelder T., Nunes N., Pico A.R., Evelo C.T. (2015). PathVisio 3: An extendable pathway analysis toolbox. PLoS Comput. Biol..

